# The Blurred Line between Form and Process: A Comparison of Stream Channel Classification Frameworks

**DOI:** 10.1371/journal.pone.0150293

**Published:** 2016-03-16

**Authors:** Alan Kasprak, Nate Hough-Snee, Tim Beechie, Nicolaas Bouwes, Gary Brierley, Reid Camp, Kirstie Fryirs, Hiroo Imaki, Martha Jensen, Gary O’Brien, David Rosgen, Joseph Wheaton

**Affiliations:** 1 Department of Watershed Sciences, Utah State University, Logan, UT 84322–5210, United States of America; 2 Ecology Center, Utah State University, Logan, UT, 84322–5210, United States of America; 3 Fish Ecology Division, Northwest Fisheries Science Center, National Marine Fisheries Service, National Oceanic and Atmospheric Administration, Seattle, WA 98112, United States of America; 4 Eco Logical Research, Providence, UT, United States of America; 5 School of Environment, University of Auckland, Auckland, New Zealand; 6 Department of Environmental Sciences, Macquarie University, Sydney, Australia; 7 Pacific Spatial Solutions, Reston, VA, United States of America; 8 Wildland Hydrology, Fort Collins, CO, 80524, United States of America; Oregon State University, UNITED STATES

## Abstract

Stream classification provides a means to understand the diversity and distribution of channels and floodplains that occur across a landscape while identifying links between geomorphic form and process. Accordingly, stream classification is frequently employed as a watershed planning, management, and restoration tool. At the same time, there has been intense debate and criticism of particular frameworks, on the grounds that these frameworks classify stream reaches based largely on their physical form, rather than direct measurements of their component hydrogeomorphic processes. Despite this debate surrounding stream classifications, and their ongoing use in watershed management, direct comparisons of channel classification frameworks are rare. Here we implement four stream classification frameworks and explore the degree to which each make inferences about hydrogeomorphic process from channel form within the Middle Fork John Day Basin, a watershed of high conservation interest within the Columbia River Basin, U.S.A. We compare the results of the River Styles Framework, Natural Channel Classification, Rosgen Classification System, and a channel form-based statistical classification at 33 field-monitored sites. We found that the four frameworks consistently classified reach types into similar groups based on each reach or segment’s dominant hydrogeomorphic elements. Where classified channel types diverged, differences could be attributed to the (a) spatial scale of input data used, (b) the requisite metrics and their order in completing a framework’s decision tree and/or, (c) whether the framework attempts to classify current or historic channel form. Divergence in framework agreement was also observed at reaches where channel planform was decoupled from valley setting. Overall, the relative agreement between frameworks indicates that criticism of individual classifications for their use of form in grouping stream channels may be overstated. These form-based criticisms may also ignore the geomorphic tenet that channel form reflects formative hydrogeomorphic processes across a given landscape.

## Introduction

The physical form of a stream channel is the result of the coupled climatic, biotic, and hydrogeomorphic processes acting upon it [1,2,3]. Accordingly, the classification of rivers into reach types based on their physical characteristics lends insight into both the formative processes that shape rivers, and the diversity of rivers that occur across an area of interest [3,4]. There are numerous frameworks for classifying streams, many of which have diverse spatial and temporal output scales (see [5,6,7]). Classification applications range from river maintenance to flood control to channel and riparian protection from land use [8], and more broadly help to disentangle natural and anthropogenic influences on channels, determine current channel condition, and forecast response to future disturbance [3]. Over the past two decades, there has been intense debate and criticism of the utility of particular frameworks [9,10,11,12,13], largely in the context of river management and restoration. These criticisms range from the limitations of a given framework at some spatial and temporal scales, to criticisms of the decisions that can arise when a framework is misapplied, to the fact that most frameworks lack measurements of process rates (e.g. sediment flux, bank migration) and process must instead be inferred from channel form. An unfortunate effect of these criticisms is that river classification frameworks, regardless of their utility, have been overlooked not for what they provide, but for perceptions of classifications’ past (mis)applications.

The discussion of individual stream classification frameworks has been subsumed in a broader conversation [12,14,15] that differentiates frameworks in terms of whether they are ‘form-based’ or ‘process-based.’ Form- based frameworks, those that classify stream reaches based on their physical attributes, are often criticized as being overly simplistic. Yet to criticize frameworks on the notion that they are ‘*only* form-based’ is to ignore a basic tenet of geomorphology: that *form implies process* [3]. That is, measurements of river form are direct reflections of the processes acting to shape that form [1,2,16,17]. Indeed, nearly all classification frameworks use metrics that describe the capacity of a channel to perform geomorphic work and adjust laterally within a valley bottom. For example, many classifications include measures of channel gradient, valley setting or entrenchment, and sediment characteristics [6,18,19,20]. The fundamental basis of geomorphology is concerned both with landforms and the processes that shape them. While the focus on quantifying process has helped geomorphology mature beyond its observational and empirical roots [21], the notion that the study of form, or inference about process from form, is inherently flawed is short-sighted. This over-simplification implies that form and process are at best, distinct, and at worst, mutually exclusive. In reality, the line between form and process is blurred, as river form and hydrogeomorphic processes are directly related.

In a long history of disagreement between proponents and detractors of particular classification frameworks, and over the relative utility of form- versus process-based classification in general, it is of note that direct comparisons of frameworks are exceedingly rare [11,22,23,24]. This may be due to the inherent difficulty in comparing methodologies that produce results at different spatial scales and which seek to describe past or present river condition. These methodologies also often require disparate types and amounts of input data and varying degrees of geomorphic expertise to complete. Nevertheless, the geomorphic community would benefit from a more clear understanding of the degree to which various river classifications, which differ in whether or how they include or infer process from form, reach similar or disparate conclusions with regard to their output [3].

This paper applies four classification frameworks across a watershed of high conservation interest in the Pacific Northwest, USA (Table 1). Each of these frameworks contains, to varying degrees, metrics that reflect the form of channels and floodplains, and/or the processes operating upon those channels and floodplains. Our goal is to perform **the first direct comparisons of classification frameworks at the watershed scale**, and in so doing, to elucidate the reasons for similarities and differences between classification outputs. Where frameworks differ in the geomorphic attributes (e.g. channel planform, bed material, valley confinement) of their output, we attempt to ascertain the methodological differences that lead to divergence in classification. Herein we focus on the *River Styles Framework* (RSF; [6]), the *Natural Channel Classification* system (NCC; developed by *Beechie and Imaki* [25]), and the *Rosgen Classification System* (RCS; [26,27]). We contrast these with an example of a flexible *statistical classification* approach that clusters field-measured, reach-scale data into channel-form-based groups (Table 1). The RSF and RCS are commonly applied in Australian and North American stream and watershed assessments, respectively. The NCC framework, as presented here, uses elements of the Montgomery and Buffington classification [20] to create a network of pre-disturbance channel types across the U.S. Pacific Northwest [20]. Statistical classification is a flexible family of approaches to grouping stream channels, and is increasingly common in geomorphology (e.g. [28]), hydrology (e.g. [29]) and ecology (e.g. [30]) for identifying patterns among observations. These frameworks have been selected because of (a) their popularity in the geomorphology and aquatic habitat communities, (b) the wide spatiotemporal range of their outputs, and (c) the varying degrees to which they directly or indirectly account for processes operating on river systems (Table 1).

**Table 1:** Summaries of the four classification frameworks applied to streams of the Middle Fork John Day River Basin: River Styles, Natural Channel Classification, Rosgen Classification System, and statistical classification.

Classification Framework (abbreviation)	Description	Examples	Data requirements	Classified output	References
River Styles Framework (RSF)	A hierarchical, multi-scale classification scheme for describing river character and behavior. River Styles can be used to understand river condition, recovery potential and prioritize management.	Use in river management practice across NSW, Australia [6,31,32]; Correlates to downstream sediment storage and landscape connectivity [33,34,35,36,37]; Ecological community composition varies as a function of River Styles [38,39]	Field, remote-sensing and other GIS data on geology, hydrology, and stream geomorphic setting to identify broad-scale to local controls on river character and behavior.	Continuous stream network (NHD+)	[6,31,32,40]
Columbia Basin Natural Channel Classification (NCC)	NCC is a model-based stream classification using a machine-learning (support vector machine) algorithm to group reaches based on their historic, undisturbed planform. Divides reaches into groups based on channel width before sub-dividing on reach-level remote sensing data.	A historic planform map and dataset for the Columbia River Basin [25]	Remotely-sensed channel slope, discharge, valley confinement, sediment supply, and sediment size are used as predictors of channel planform in a modeling framework.	Continuous, pre-disturbance stream network (NHD)	[25]
Rosgen Classification System (RCS)	RCS is a stream-reach taxonomy based on field-collected empirical data that classifies geomorphic stream features to identify stream types by numerically bounded physical metrics. This is arguably the most commonly used stream classification system in North America and the world.	RCS can be employed to successfully restore a reach to a reference condition, provided that the reference reach is stable [41]; RCS stream type classifications provide inferences into the sensitivity of stream reaches to natural channel changes [42]	Valley morphology for broad context, and reach-scale monitoring data to calculate basic dimensionless metrics linking form to physical processes.	Individual reaches within a stream network (field-monitored reaches)	[26,27]
Statistical Classification (SC)	Statistical classification refers to any classification methods used to differentiate or group stream reaches, watersheds, etc. based on multiple physical, chemical, and/or biological attributes. Attributes are often selected for their role in driving or responding to dominant processes within a catchment.	Comparing restored, forested, and urban channels [43]; Identifying vegetation communities and environmental filters [30]; classification of desert washes [28]	Requires reach-scale monitoring data for “bottom-up” classifications. Requires remote sensing and GIS data to classify reaches from the “top-down” or correlate classified reaches to larger-scale environmental or physical processes.	Individual reaches within a stream network (field-monitored reaches). Can be applied to networks if inputs are available for stream segments or networks.	[3,28,30]

## Methods

**Fig 1:**
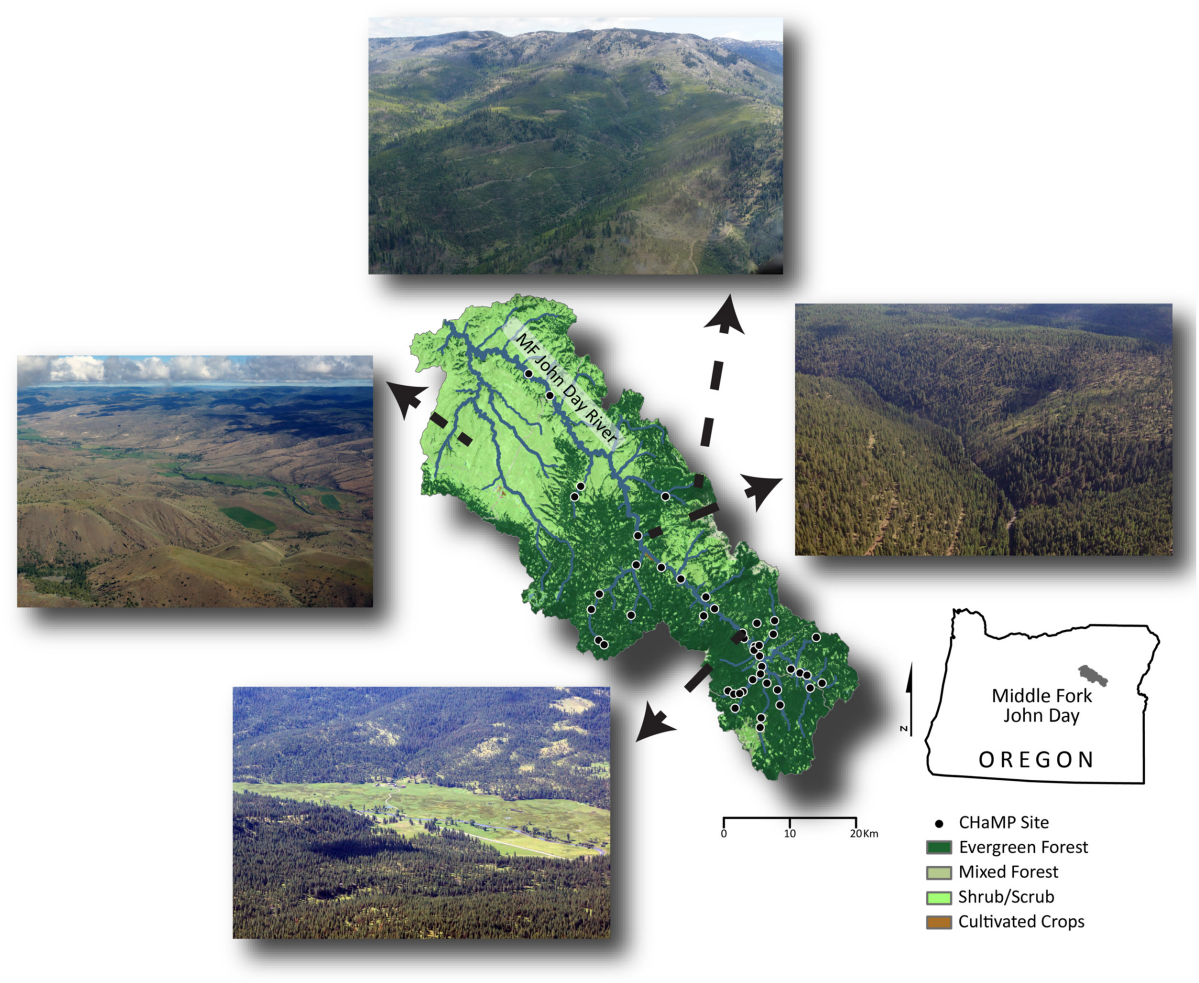
Map of the Middle Fork John Day Basin, Oregon, USA. The 33 Columbia Habitat Monitoring Program (CHaMP) reaches monitored between 2012–2013 are shown in circles. The National Landcover Dataset is presented as the base map to illustrate biophysical gradients across the watershed. Four photos illustrate the diversity of landscapes encountered across the basin.

The Middle Fork of the John Day River (MFJD; Oregon, USA) is 117 km long and drains 2051 km^2^ within the broader Columbia River Basin (Fig 1). The MFJD watershed was chosen for this research both for its physiographic diversity and due to the wealth of stream data available there, largely as a result of ongoing watershed monitoring aimed at understanding physical factors limiting salmonid population resilience (Section 2.2). These data enabled completion of the four classification frameworks herein (Table 1; Sections 2.3–2.6).

### Landscape, hydrologic, and ecological setting

The Middle Fork John Day Basin is largely composed of metamorphic and igneous rocks underlain by basalt and older extrusive rock, which have been uplifted and reworked to create a watershed marked by steep-sloped canyons, deeply dissected highlands, dissected tablelands, and rounded uplands containing broad meadows. The watershed is generally semi-arid, receiving 560 mm of annual precipitation throughout the basin on average [44]. However, the MFJD basin is also marked by a distinct elevation-dependent precipitation gradient: the upper 10% of elevations receive an average of 880 mm of precipitation, while the lowest 10% receive 370 mm. Average annual streamflow measured at the Ritter, Oregon gauging station (USGS #14044000, *A*_*d*_ = 1334 km^2^; 83 years of record) is 7.4 m^3^s^-1^.

### Salmonid conservation and watershed monitoring

Reductions in native fish populations throughout the Columbia River Basin, including the MFJD, have led to large-scale aquatic habitat monitoring across the region. In particular, steelhead trout (*Oncorhynchus mykiss*), listed as threatened under the U.S. Endangered Species Act, have seen drastic reductions in the size of their runs [45] as a direct effect of anthropogenic habitat degradation, in part due to hydropower development, land use change, and direct channel modification such as loss of large woody debris [46]. As a result, watersheds throughout the Columbia River Basin have received intensive monitoring efforts to document the status and trend of fluvial habitats that support salmonid populations [47,48].

The MFJD is monitored as part of the Columbia Habitat Monitoring Program (CHaMP; http://www.champmonitoring.org). CHaMP data, which are used to complete the four classification frameworks, are collected at wadeable, perennial streams throughout the Columbia River Basin [48]. Reaches were selected for sampling using a generalized random tessellation stratified sampling design to prevent spatial sampling bias [49]. Here we use CHaMP data from the MFJD watershed collected during 2012 and 2013 (n = 33 sites) describing channel bankfull width and depth, gradient, substrate, and sinuosity. CHaMP sampling reaches are twenty times as long as the bankfull channel width at each site (120–360 m in length).

### The River Styles Framework

The River Styles framework (Table 1) seeks to provide a “coherent set of procedural guidelines with which to document the geomorphic structure and function of rivers, and appraise patterns of river types and their biophysical linkages in a catchment context” [6]. In practice, the RSF offers the potential for a process-based, watershed-scale classification system for rivers. The RSF consists of four distinct stages that progress from (1) classifying landscapes and current river form and function, to (2) assessing geomorphic river condition in context of reach evolution, to (3) understanding and forecasting trajectories of river change, and (4) prioritizing catchment management. A full description of the methods entailed in the RSF can be found in [6]. Here we describe the application of stage one of the RSF, which has been completed for the MFJD as part of an ongoing effort to contextualize site-specific CHaMP monitoring data in a watershed setting [50]. Stage one provides a baseline assessment of current reach types (referred to as ‘river styles’) in a system with emphasis on longitudinal variability of river form (i.e. longitudinal profile analyses) along the mainstem channel and tributary network.

The RSF begins with the classification of landscape units (Fig A in S1 File), each of which contain a unique distribution of river styles. Within a given landscape unit, stream reaches are classified based on their valley confinement, presence or absence of floodplains, channel planform, distribution of in-channel and floodplain geomorphic units, and dominant channel substrate (Table 2). In contrast to the other classification systems presented here and those used among practitioners (e.g. [20,26]), there is no intrinsic limit on the number of river styles that may occur in a watershed of interest. In practice, once the diversity of river styles for a particular watershed is known, a river styles tree (Figs B–D in S1 File) can be constructed that allows for the classification of any stream segment from those found in the watershed. The top-level discriminator in the RSF is valley confinement (Figs B–D in S1 File), which Brierley and Fryirs [6] define as “the proportion of the channel length that abuts a confining margin on either side” [6].

**Table 2:** Form-based channel metrics included in classification analyses.

Metric of channel form	Relationship to channel processes	RSF	NCC	RCS	Clustering
Valley confinement	Ability for lateral adjustment [6,61]; Supply of sediment and vegetation [25,56,65]	**X**	**X**	**X**	
Presence or absence of channels	Magnitude/frequency of flow [6]; Presence of hyporheic flow [6]; Valley sedimentation/filling [31]	**X**		**X**	
Number of channels	Accommodation space for flow [6]	**X**		**X**	
Distribution of floodplains	Accommodation space for flow [40]; Influence of vegetation on flow [61]	**X**		**X**	
Gradient or channel slope	Transport competence via stream power [57,58,59,60]	**X**	**X**	**X**	**X**
Sinuosity	Competence via slope/stream power [62,63]; Ability for lateral adjustment [64]; Input of vegetation/sediment from banks [65]	**X**	**X**	**X**	**X**
Lateral channel stability	Ability for lateral adjustment [66]; Input of vegetation/sediment [65,66,67]	**X**			
Unit stream power	Transport competence [57,58,59,60]	**X**			
Site discharge	Transport competence via stream power [57,58,59,60]		**X**	**X**	
Bankfull width	Transport competence via unit stream power [57,58,59,60]		**X**	**X**	**X**
Wetted width	Transport competence via stream power [57,58,59,60]				**X**
Bankfull depth	Transport competence [62,63]			**X**	
Width: depth ratio	Transport Competence [68]; Influence of Vegetation on Flow [69]			**X**	**X**
Entrenchment ratio	Accommodation space for flow [61]; Transport competence via stream power [57,58,59,60]; Ability for lateral adjustment [70]; Input of sediment and vegetation [25,56,65]			**X**	
Bed material (categorical)	Transport competence [62,63]	**X**			
*D*_*16*_, *D*_*50*_, *D*_*84*_	Transport competence [62,63]			**X**	**X**
Geomorphic units (channel and floodplain)	Transport competence [71]; Transport regime [72]; Magnitude/duration/frequency of flooding [6,40,57,72]; Influence of vegetation on flow [73,74]	**X**			

Note that inclusion of metrics in each classification framework reflects only the stages that were completed in this research, and that ‘processes’ only include geomorphic dynamics, and exclude ecological processes.

We used O’Brien and Wheaton’s [50] delineation of river styles for the MFJD where the boundaries between landscape units were defined using remote sensing data including elevation (10 m and 1 m digital elevation models; [51]), slope, underlying geology [52], dominant vegetation [53], and Level IV EcoRegion boundaries [54]. Following the delineation of landscape units, individual river styles were initially digitized on the National Hydrography Dataset (NHD; as polylines in ArcGIS; ESRI, Redlands, CA) using aerial photos ([55]; 1 m resolution) and elevation datasets as a guide. O’Brien and Wheaton [50] conducted field visits in the summer of 2012 and 2013 to confirm the accuracy of these delineations, refine the distinguishing characteristics of each river style and its location in the river style tree (Figs B–D in S1 File) and pinpoint boundaries between river styles when they could not be delineated using remote sensing data.

A hierarchical framework, components of the RSF can be considered both form- and process-based (Table 2). Individual River Styles are classified in part by their behavior, that is, interpreting how instream and floodplain geomorphic features (landforms) are created and reworked under various flow regimes. This interpretation is field-checked via geomorphic mapping during visits to sites [50]. The initial differentiation of reaches is conducted at the valley setting scale, based on valley confinement. This is analogous to Montgomery’s [56] process domains, which reflect the channel’s access to sediment sources and the mechanisms through which sediment reaches the channel (Table 2). Stream power is estimated continuously along the channel and can be used to infer reach boundaries [6]. Within each valley setting, river styles are classified based on metrics of channel form that are directly tied to geomorphic processes like stream discharge and power that govern sediment transport, along with channel planform (including the presence or absence of a channel), the array of instream and floodplain geomorphic units along the reach, and bed material texture (Table 2).

### Natural channel classification

Natural channel classification [25] seeks to predict the background, or pre-disturbance, planform of alluvial channels found in an area of interest (Table 1). To this end, Beechie and Imaki [25] constructed a probabilistic map of pre-disturbance channel planforms across the Columbia River Basin, USA (drainage area 674,500 km^2^). Channel classes identified in NCC include *confined* channels and four channel patterns for unconfined reaches: *straight*, *meandering*, *island-braided*, and *braided*. These four unconfined channel patterns are common planforms for alluvial, floodplain rivers [16,18,75], which have distinctly different morphologies, dynamics, and ecological attributes [74,76]. In NCC, confinement is defined as the ratio of bankfull width to valley width, and unconfined channels are those where the valley floor width is more than four times the bankfull width. Predictor variables in the model were based on known physical controls on channel pattern, including channel gradient, discharge, valley confinement, sediment supply, and sediment size [77]. Channel slope, discharge, and confinement were estimated from digital elevation models. Relative reach slope, percent of the watershed in unvegetated alpine terrain, and percent of the watershed in fine-grained erosive sediments were hypothesized to be surrogates for sediment supply and size, respectively. Relative slope is the slope of a reach minus the slope of its upstream neighbor. Positive relative slope values indicate that a reach is steeper than its upstream neighbor (likely sediment supply limited or undersupplied), and for a given slope and discharge is likely be narrower, deeper, and more armored [18,78], whereas negative values indicate that a reach is more likely to have low transport capacity relative to bed load supply (i.e., transport limited or oversupplied), and will likely be wider, shallower, and finer grained or less armored.

For all channel segments with bankfull width > 8 m, attributes were assigned to each 200-m long reach in the study area (> 2,000,000 reaches) based on available geospatial data, and adjacent reaches with similar characteristics were then aggregated into sets of geomorphically meaningful reaches. A sample of more than 30 relatively natural reaches of each channel pattern was selected as the training data set (i.e., the natural channel pattern was not obscured by contemporary land use or dams). Hence, the model should predict channel patterns *expected in the absence of human impacts*, *rather than current channel form*. A support vector machine (SVM) classifier was used to relate all 63 possible combinations of reach attributes to channel pattern using a total training data set of 147 reaches. The multiple models were cross-validated for classification accuracy, and the most accurate SVM model was then used to predict channel pattern for all reaches in the study area. Bootstrapping of the final model created 1000 separate predictions of channel pattern for each reach, and the consistency of predictions can be used as an indicator of model uncertainty for each reach. For example, if 85% of the predictions for a reach were ‘braided,’ we considered that reach to have a high likelihood of having a braided channel pattern. This statistical approach produces maps of (1) the most likely channel pattern for each reach in the Columbia River Basin, and (2) uncertainty in the channel pattern prediction. For channels with bankfull width < 8 m, reaches were classified based on gradient [20] as pool-riffle (slope < 0.02), plane-bed (slope between 0.02 and 0.03), step-pool (slope between 0.03 and 0.08) or cascade (slope > 0.08).

Like the RSF, NCC contains elements based in process and form. NCC uses basin-scale measurements of land cover and surficial geology to estimate sediment supply, along with estimated valley confinement, the combination of which reflects sediment delivery to channels [56]. In addition, remotely sensed measurements of channel form (i.e. channel width and gradient) reflect the ability of a reach to transport supplied sediment [60,79,80]. Together, these can be used to estimate the form of a given reach under baseline conditions.

### Rosgen classification system

The Rosgen Classification System (RCS; [26,81]) provides a standardized workflow for river classification based on a field survey of the geomorphic characteristics of a particular stream reach (Table 1). RCS consists of four hierarchical stages of classification moving from coarse to fine spatial scales [27]. In Level I, the system uses spatial data describing valley confinement, channel planform, local soil types, hydrologic regime, and watershed physiography to establish a broad geomorphic characterization of river reaches. In Level II, the geomorphic characteristics of a site (e.g. entrenchment ratio, width/depth ratio, sinuosity, median grain size, and gradient) are assessed and a particular stream type is assigned to the reach using the decision tree first presented by Rosgen [26]. Like the RSF and NCC, in Level II the RCS emphasizes valley setting and confinement early in the process. RCS uses a field-measured entrenchment ratio (channel wetted width at two times bankfull depth divided by the bankfull width), which is analogous to the bankfull to valley width ratio that NCC uses as a proxy for confinement. In Level III, the stream’s condition is assessed based on channel planform, bed and bank stability, occurrence and type of riparian vegetation, and any alterations in flow regime. Finally, stream types delineated in Levels II and III are field-checked by direct measurements of sediment transport and size, flow, bed/bank stability, and rates of bank erosion to ensure a valid stream type classification has been made (Level IV).

We classified the 33 CHaMP reaches in the Middle Fork John Day Basin (Fig 1) using Levels I and II of the RCS. We used DEMs (10 m and 0.1 m grid resolution), aerial imagery (1 m resolution), and ground-based assessments to infer the Level I valley types surrounding each CHaMP reach. Delineation of bankfull elevation was completed by trained technicians in the field and surveyed as part of the CHaMP topographic survey. Calculations of width-to-depth ratio, channel sinuosity, entrenchment ratio, and channel gradient were derived from CHaMP topographic survey DEMs (0.1 m grid resolution) using the River Bathymetry Toolkit (RBT; [82]). A bankfull water surface was derived by detrending a DEM and best-fitting a water stage through the measured bankfull points and examining inflections in the hydraulic geometry using the CHaMP Topo Toolbar (https://sites.google.com/a/northarrowresearch.com/champtools/). Measurements that typically are derived from cross sections using RCS were derived from averages of 100+ cross sections spaced at 1-meter intervals at every CHaMP site and processed using the RBT. These metrics allowed us to categorize each CHaMP reach into broad level RCS stream types (A-G). By combining broad RCS stream types with median grain size data (*D*_*50*_) collected during CHaMP surveys, we classified each site into a final channel type according to the RCS classification. Although we did not explicitly validate our reach type delineations in the field (e.g. Level IV as described above), the wealth of on-the-ground photographs and high-resolution topographic data (0.1 m-resolution DEMs) collected as part of CHaMP surveys were used to ensure the validity of classified reaches.

Level II of the RCS is a form-based approach, relying on measurements of channel geometry and bed material size to classify stream reaches (Table 2; [27]). It has received criticism in the geomorphic literature for its methods, more so than the other classification frameworks used herein [12,14,15], on the assertion that distinctions between stream types may not represent a distinct suite of processes, but rather simply reflect different points along a process continuum [10]. We would argue, however, that this latter criticism may be an inherent drawback to nearly all hierarchical classification frameworks [3,20]. At the same time, the RCS, like the other classification frameworks used here, relies on measurements of channel form as surrogates for geomorphic process, and perhaps more so than the other three approaches, requires direct field-based measurements to do so.

### Statistical classification

Multivariate statistical classification provides a flexible framework to identify patterns between reaches based on channel form and/or landscape setting (Table 1). Multivariate statistical approaches, including hierarchical clustering, use distance measures to group stream reaches based on their similarity (or dissimilarity) across multiple physical attributes [83]. Statistical classification is a family of techniques, rather than a single technique, allowing flexibility in the input data used, the distance measure used to compare similarity across observations, and in the case of clustering, the algorithm used to identify meaningful groups of observations [83]. Here we provide an example of how these techniques can be employed in the same capacity as the other stream classifications used herein.

We classified the 33 CHaMP sites in the Middle Fork John Day Basin by clustering reaches on multiple instream geomorphic attributes: bankfull width, wetted width, site sinuosity, stream gradient, bankfull width to depth ratio, and *D*_*16*_, *D*_*50*_, and *D*_*84*_ particle size. CHaMP metrics that reflect sediment size and channel form were selected in order to maintain consistency with data used in the classifications presented in Sections 2.3, 2.4, and 2.5. We selected a partitioning around medoids clustering algorithm in R (‘cluster’ package; [84, 85]), a divisive clustering technique, to identify groups of distinct reach types based on the Euclidean distance between reaches’ instream geomorphic attributes. We validated differences in stream attributes between reach clusters using PERMANOVA [86]. We plotted the cluster solution within a principal components analysis (PCA) of the same stream channel attributes, visually comparing CHaMP reaches classified under each method (RSF, NCC, RCS, clustering). Full statistical methods and results are presented in the supporting information (Text A in S1 File).

The statistical classification applied here is purely form-based, incorporating geomorphic process only by grouping channels on their physical attributes’ similarity (Table 2). Field-derived measurements of channel gradient, bankfull channel dimensions, and bed material size were used to describe channel form, which, in aggregate, reflect the ability of a given stream reach to transport supplied sediment, similar to how RCS estimates process using form-based attributes (Section 2.5). An important distinction between the statistical classification and the other three classifications used here is how they incorporate valley setting. While RSF, NCC, and RCS estimate sediment supply and delivery processes by classifying valley setting (albeit at a later stage in RCS), the statistical clustering employed here does not use valley confinement or surrogates (stream order, valley slope) as a discriminator in its classification.

Statistical clustering approaches are relatively rare in geomorphic channel classification compared to the other three frameworks described here. Despite the need for further exploration of this technique, the purpose of this research was not to explore the effect of various statistical classification algorithms (e.g. agglomerative versus divisive clustering, different distance measures, etc.), but to select a parsimonious framework that aggregates channels into user-defined sets of groups based on channel metrics. Future comparisons of stream channels should build on this example by comparing multiple statistical classification methodologies in geomorphic classifications.

### Assessing classification framework agreement

To compare agreement between classification frameworks at the 33 CHaMP sites discussed in Section 2.2, we compared classification outputs by using both (a) expert judgment and (b) a multivariate comparison of reaches based on their resulting outputs in each of the four frameworks.

**Fig 2:**
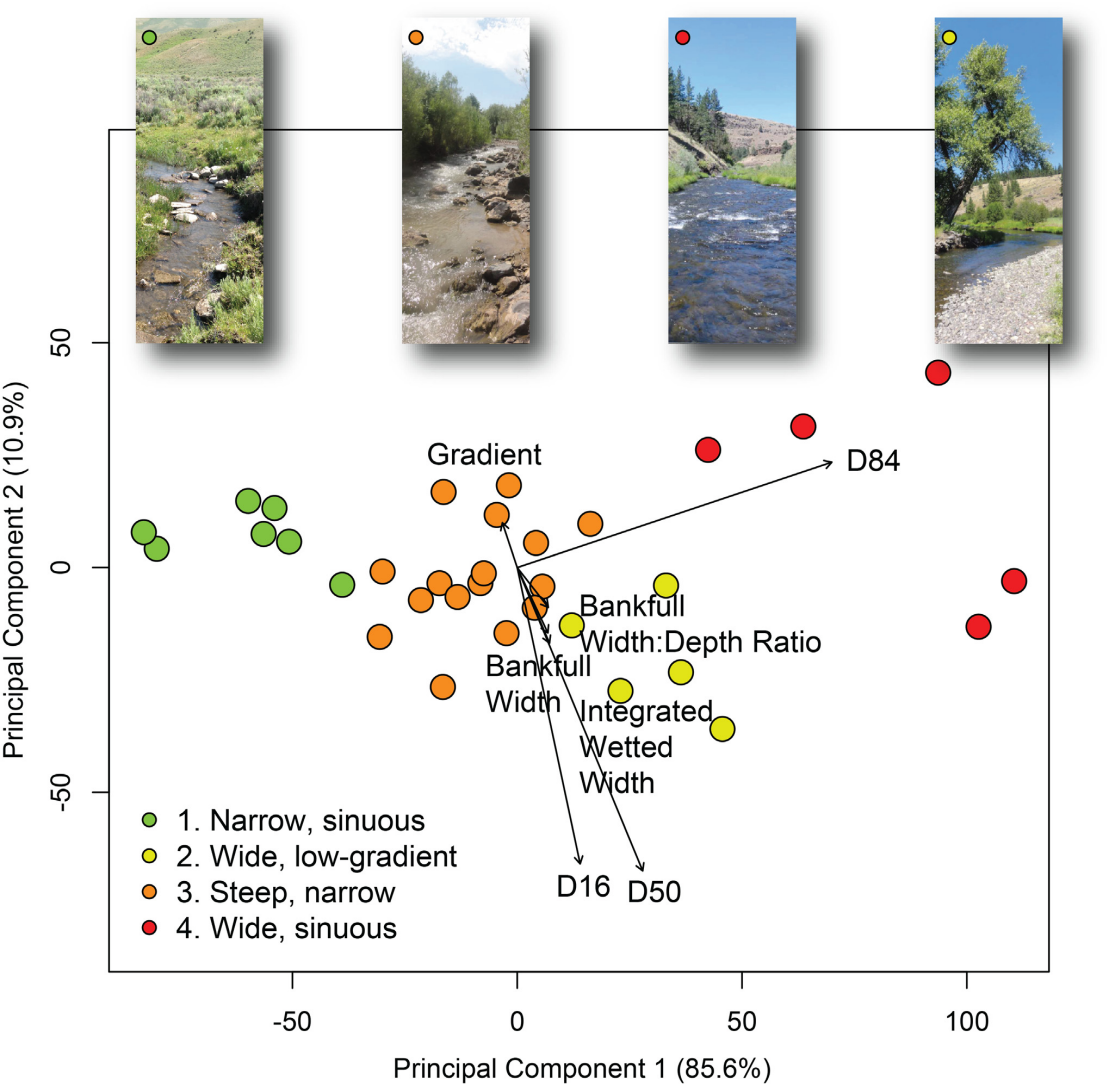
Statistical clustering of reaches using principal components analysis (PCA) based on gradient, *D*_*16*_, *D*_*50*_, *D*_*84*_, bankfull width, bankfull width:depth ratio, and integrated wetted width (i.e. channel width at time of sampling), classified into four discrete groups using partitioning around medoids. Vectors of stream channel variables are plotted based on the strength of their correlation to the PCA (e.g. longer vectors are more strongly correlated to the channel form variable PCA). The first and second principal components explained 85.6% and 10.9% of the variability in the reach attribute data within the PCA. Point colors represent which cluster each reach was classified into, and representative photographs provide examples of characteristic reach morphology for each cluster.

To compare the frameworks’ outputs using an expert judgment approach, we began by using the eight reach types identified by Natural Channel Classification, as these classes provided intuitive and straightforward descriptors of channel planform [25]. For each NCC reach type, we identified the most closely related reach types from the RSF, the RCS (using top-level channel types A-G), and statistical clustering. Where available (RSF, RCS), decision trees were used to select those reach types that best approximated each NCC type based on common geomorphic metrics (gradient, geomorphic units present, planform). In the case of statistical clustering, the geomorphic attributes inherent to each of the four clusters (Fig 2) were used to approximate the corresponding NCC reach type.

Those RSF, RCS, and statistical clustering reach types that were most closely related to each NCC type were classified as being in “good” agreement (e.g. all geomorphic attributes of the reach type could conceivably be present in the associated NCC channel class), while those which were only marginally related to each NCC class (that is, some aspects of the reach types fit with an NCC class while others did not) were classified as having “moderate” agreement (Table 3). RSF, RCS, and clustering reach types with no characteristics in common with NCC classes were classified as having “poor” agreement. While this method is inherently qualitative, we attempted to take an inclusive approach when determining agreement among reach types between frameworks, as considerable geomorphic variability can exist across each reach type within a given framework [6,27].

**Table 3:** Analogous reach types between NCC, RSF, RCS, and statistical clustering based on common geomorphic attributes. Those reach types with good (G) or moderate (M) agreement are included, while those with poor agreement are not shown here, but are noted in Table 4.

NCC reach type	RSF reach type	RCS reach type	Statistical cluster
Island Braided	Low Sinuosity Planform Controlled Anabranching (G); Intact Valley Fill (M); Alluvial Fan (M)	D (G)	2: Wide, Sinuous (M)
Meandering	Meandering Gravel Bed (G); Meandering Planform-Controlled Discontinuous Floodplain (G); Low-Moderate Sinuosity Gravel Bed (M); Low-Moderate Sinuosity Planform-Controlled Disc. Floodplain (M); Bedrock-Controlled Elongate Discontinuous Floodplain (M); Low-Moderate Sinuosity Gravel Bed (M)	C (G), E (G), G (M), F (M)	4: Wide, Sinuous (G); 1: Narrow, Sinuous (M); 2: Wide, Low-Gradient (M)
Straight	Boulder Bed (G); Meandering Planform-Controlled Disc. Floodplain (G); Confined Valley—Floodplain Pockets (G); Low-Moderate Sinuosity Partly Confined Disc. Floodplain (G); Low-Moderate Sinuosity Gravel Bed (G); Alluvial Fan (M); Bedrock-Controlled Elongate Discontinuous Floodplain (M)	A (G); B (G); G (M)	2: Wide, Low-Gradient (G); 3: Steep, Narrow (G)
Confined	Entrenched Bedrock Canyon (G); Confined Valley—Floodplain Pockets (G); Step Cascade (G); Steep Perennial Headwater (M); Steep Ephemeral Hillslope (M)	A (G); F (G); G (G); B (M)	1: Narrow, Sinuous (G); 3: Steep, Narrow (G); 2: Wide, Low Gradient (M)
Cascade	Step Cascade (G); Boulder Bed (G); Floodplain Pockets (M); Steep Perennial Headwater (M); Steep Ephemeral Hillslope (M)	B (G); F (G); G (G); A (M)	3: Steep, Narrow (G) 1: Narrow, Sinuous (M)
Pool Riffle	Meandering Gravel Bed (G); Meandering Planform Controlled Discontinuous Floodplain (G); Confined Valley—Floodplain Pockets (G); Bedrock-Controlled Elongate Discontinuous Floodplain (G); Low-Moderate Sinuosity Planform Controlled Disc. Floodplain (M); Meandering Partly-Confined Floodplain (M)	C (G); F (G); G (G); E (G); B (M)	1: Narrow, Sinuous (G); 2: Wide, Low Gradient (G); 4: Wide, Sinuous (M)
Step Pool	Boulder Bed (G); Step Cascade (G); Steep Perennial Headwater (G); Steep Ephemeral Hillslope (G); Confined Valley—Floodplain Pockets (M)	B (G); F (G); G (G); A (M)	3: Steep, Narrow (G); 1: Narrow, Sinuous (M)
Plane Bed	Entrenched Bedrock Canyon (G); Confined Valley—Floodplain Pockets (G); Bedrock Controlled Elongate Discontinuous Floodplain (G); Low-Moderate Sinuosity Planform Controlled Disc. Floodplain (G); Meandering Planform Controlled Floodplain (M); Boulder Bed (M); Steep Perennial Headwater (M); Steep Ephemeral Hillslope (M)	A (G); B (G); C (G); F (G); G (G)	3: Steep, Narrow (G); 1: Narrow, Sinuous (M); 4: Wide, Sinuous (M)

In addition to our expert judgment-based, qualitative comparison of framework outputs, we also quantitatively assessed reaches’ classification output agreement using a multivariate approach. We created a table of each reach’s classification outputs, calculated a Gower’s dissimilarity matrix between each reach, and then visualized reaches in a cluster analysis and ordination. Gower’s distance scales nominal variables between 0 and 1, allowing us to calculate the similarity of discrete reaches’ agreement using the categorical outputs from each framework. We clustered reaches based on their output dissimilarity using an average linkage clustering algorithm. We then conducted a two-dimensional principal coordinates analysis (PCoA) on the same Gower’s dissimilarity matrix of classification outputs, and used this to visualize similarity between classifications at each reach. It is important to note that while the qualitative, expert judgment-based approach above uses NCC as the top-level discriminator in assessing framework agreement, the statistical assessment described here is a multivariate comparison of outputs between all four frameworks.

## Results

### The River Styles framework

**Fig 3:**
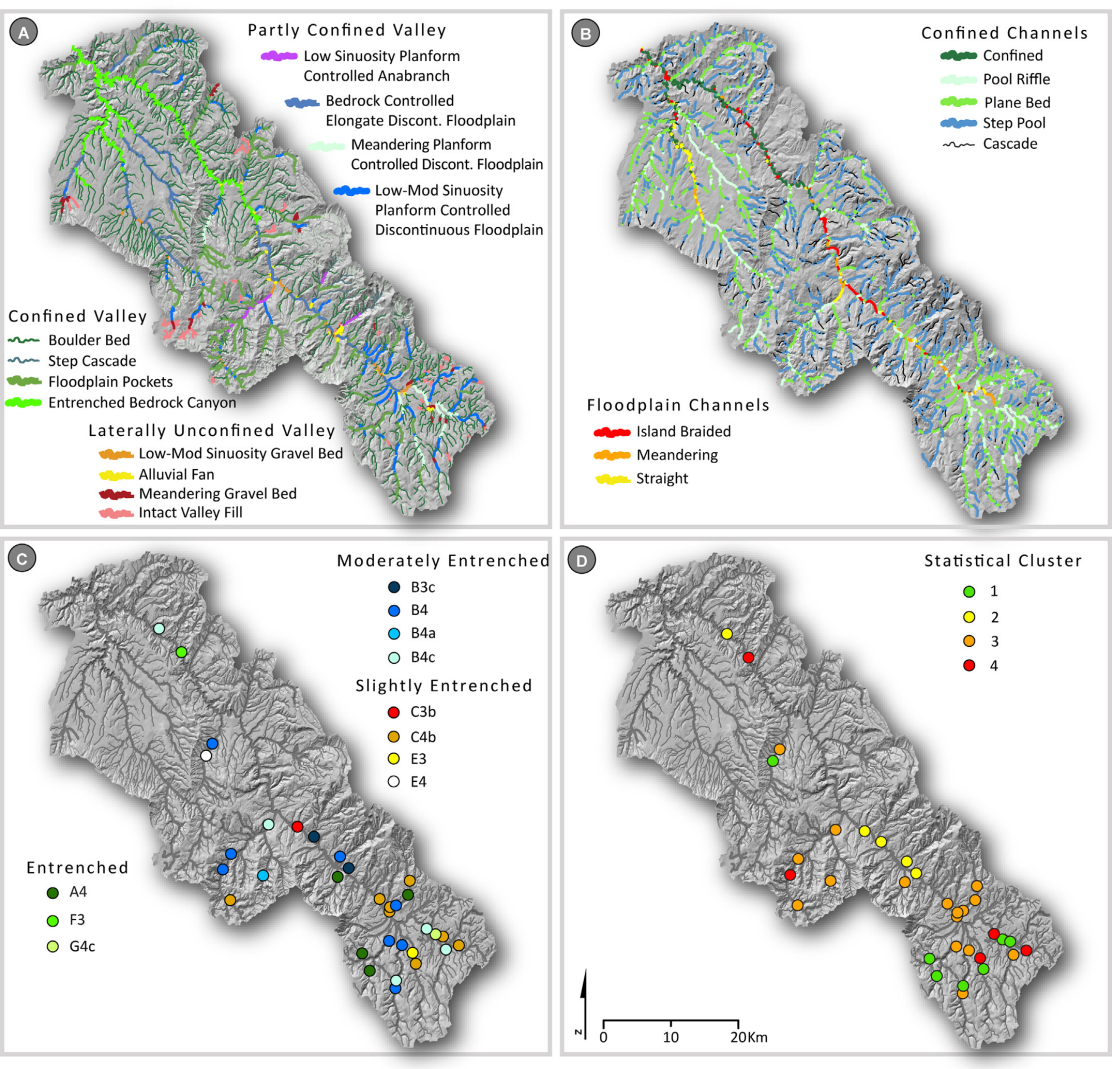
Results of the four classifications. (A) River Styles, (B) Natural Channel Classes, (C) Rosgen Classification System, and (D) statistical classification with clustering (partitioning around medoids) mapped across the Middle Fork John Day Basin. River Styles and Natural Channel Classes are mapped across the entire stream network, while Rosgen Classification System and statistical classification results are presented only for CHaMP reaches. Full River Style and Natural Channel Class results for CHaMP reaches are presented in Table 4.

In total, 14 distinct river styles were classified across the MFJD Watershed. To begin, landscape units were classified across the watershed (Fig A in S1 File). The river styles trees showing the characteristics of each river style are shown in Figs B–D in S1 File, and the distribution of river styles within the MFJD Watershed is shown in Fig 3A, with distinctions made based on valley confinement (confined, partly confined, laterally unconfined; [34]). Overall, confined valley channels were the most common river styles across the MFJD Watershed (86% of total stream length; Table 4), whereas channels in partly confined valleys (8%) and laterally unconfined valleys (6%) were far less common (Fig 3A). Small, low-order, confined channels (boulder bed and steep ephemeral hillslope river styles) comprised the majority of total stream length within the watershed (68%, Table 4). Regarding the most common classifications of CHaMP sites, 33% of sites were classified as partly confined valley with low-moderate sinuosity planform-controlled discontinuous floodplain reach types, 15% were classified as confined valley with occasional floodplain pockets, and 12% each were classified as partly confined valley with meandering planform-controlled discontinuous floodplain and bedrock-controlled elongate discontinuous floodplain reach types (Fig 3A; Table 4).

**Table 4:** Classification results for the four methods compared here.

Classification framework	Confinement	Reach type	Total stream length (km)	% Total length	# CHaMP reaches	% CHaMP reaches
RSF	Confined	Boulder bed	1230.7	30.2	1	3.0
RSF	Confined	Entrenched bedrock canyon	121.1	3.0	2	6.1
RSF	Confined	Occasional floodplain pockets	242.5	6.0	5	15.2
RSF	Confined	Step cascade	37.9	0.9	0	0
RSF	Confined	Steep ephemeral hillslope	1542.3	37.9	0	0
RSF	Confined	Steep perennial headwater	319.4	7.8	0	0
RSF	Partly confined	Meandering planform controlled discontinuous floodplain	34.5	0.8	4	12.1
RSF	Partly confined	Low sinuosity planform controlled anabranching	18.2	0.5	2	6.1
RSF	Partly confined	Low-moderate sinuosity planform-controlled discontinuous floodplain	170.2	4.2	11	33.3
RSF	Partly confined	Bedrock controlled elongate discontinuous floodplain	113.8	2.8	4	12.1
RSF	Laterally unconfined	Low-moderate sinuosity gravel bed	31.9	0.8	1	3.0
RSF	Laterally unconfined	Alluvial fan	49.3	1.2	1	3.0
RSF	Laterally unconfined	Meandering gravel bed	62.9	1.5	2	6.1
RSF	Laterally unconfined	Intact valley fill	99.4	2.4	0	0
NCC	Bankfull width > 8 m	Straight	132.9	7.8	8	24.2
NCC	Bankfull width > 8 m	Meandering	34.7	2.0	3	9.1
NCC	Bankfull width > 8 m	Island-braided	42.8	2.5	2	6.1
NCC	Bankfull width > 8 m	Confined	76.5	4.5	3	9.1
NCC	Bankfull width < 8 m	Pool riffle	129.9	7.7	5	15.2
NCC	Bankfull width < 8 m	Plane bed	431.5	25.4	8	24.2
NCC	Bankfull width < 8 m	Step pool	595.3	35.1	4	12.1
NCC	Bankfull width < 8 m	Cascade	253.7	14.9	0	0
RCS	Entrenched	A4			4	12.1
RCS	Entrenched	F3			1	3.0
RCS	Entrenched	G4c			1	3.0
RCS	Moderately entrenched	B3c			2	6.1
RCS	Moderately entrenched	B4			8	24.2
RCS	Moderately entrenched	B4a			1	3.0
RCS	Moderately entrenched	B4c			5	15.2
RCS	Slightly Entrenched	C3b			1	3.0
RCS	Slightly Entrenched	C4b			8	24.2
RCS	Slightly Entrenched	E3			1	3.0
RCS	Slightly Entrenched	E4			1	3.0
Clustering		Narrow, sinuous (1)			7	21.2
Clustering		Wide, low-gradient (2)			5	15.2
Clustering		High-gradient, narrow (3)			16	48.5
Clustering		Wide, sinuous (4)			5	15.2

River Styles and Columbia Basin Natural Channel Classification are summarized across the entire network and at CHaMP sites, while the Rosgen Classification System and clustering classifications are summarized only for reaches with CHaMP channel data.

### Natural channel classification

**Fig 4:**
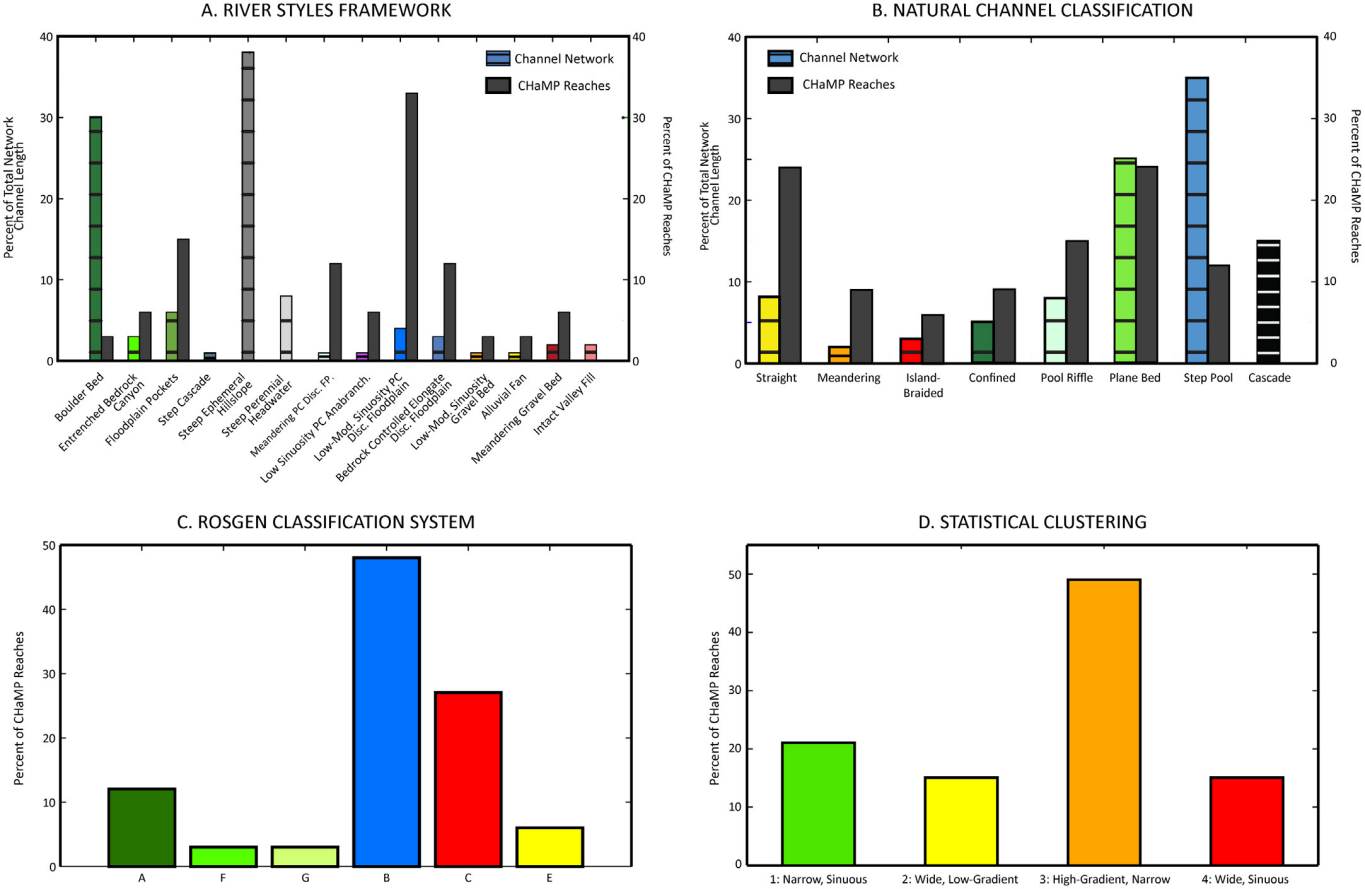
Classification results across network and sites. Percent of total network channel length and percent of CHaMP sites classified into reach types using each classification framework (A-D).

Natural Channel Classification derived nine channel patterns across the Columbia River Basin [25], eight of which were predicted within the MFJD Watershed (Figs 3B and 4B). By total stream length, the majority of reaches (83%) were small channels with bankfull width < 8 m (Table 4). Across the MFJD, 35% of the total reach length was classified as step-pool channels, and 25% classified as plane-bed channels [20]. For channels > 8 m bankfull width, 8% of the total reach length was classified as having a straight planform, 3% of channels classified as island-braided, and 2% classified as meandering (Fig 3B; Table 4). The remaining reaches >8 m were classified as confined channels because valley width was less than four times bankfull channel width [25]. With regard to the most common classifications of CHaMP sites, 25% of sites each were classified as straight or plane bed reaches, with an additional 15% of sites classified as pool riffle (Fig 3B; Table 4).

### Rosgen classification system

We classified 11 RCS stream types within 33 CHaMP reaches in the MFJD Watershed (Fig 3C; Table 4). The most common stream types, each containing 24% of the CHaMP reaches, were B4 (stable plane bed with occasional pools) and C4b (low gradient, meandering, riffle/pool sequences; Fig 4C). In total, 50% of the reaches were B stream types, all of which were within valley type II (colluvial, moderately steep and confined), with a single exception. C stream types (sinuous, wide and low-gradient) were the next most common (27%) and E (highly sinuous, coarse-fine bed), F (entrenched, wide, moderately sinuous, low gradient), and G (entrenched, low-gradient, low width:depth ratio) types were the least common (3% each). Only one CHaMP site had a substantial length of side channels (24%), however the other metrics did not fit a D stream type. Therefore, we did not delineate any multi-threaded channels (RCS stream type D).

### Statistical classification

Because statistical clustering does not test for an *a priori* set of outcomes as other statistical approaches might, we compared multiple clustering results (two to ten clusters of channels) from clusters generated using a partitioning around medoids algorithm. We selected a four cluster final solution based on cluster fidelity; that is, the statistical and geomorphic differences in the multiple attributes used to distinguish between groups, minimizing overlap between cluster groups (Fig 2; Tables A–C in S1 File). We did this objectively rather than trying to create an *a priori* number of reach types to match the other frameworks’ number of outputs. After plotting the final cluster solution within a principal component analysis (PCA), the clustered stream channel attributes showed that each group differed based on multiple channel form attributes. The PCA indicated that the four identified clusters were meaningful representations of the sampled reaches and not just statistical artifacts. Each cluster was named based on the dominant attributes that differentiated clusters from one another. The four final groups consisted of (1) narrow, sinuous, high-gradient reaches (n = 7), (2) wide, low-gradient, coarse substrate reaches with high width to depth ratios (n = 5), (3) high-gradient, narrow reaches with moderate-sized substrates (n = 16), and (4) moderate gradient, wide and sinuous, coarse-substrate reaches (n = 5; Fig 2; Table 4). The number of CHaMP sites assigned to each cluster are shown in Fig 4D. Channel clusters were significantly different from one another (PERMANOVA; *p* < 0.05), and particle *D*_*16*_, _*D50*,_ and *D*_*84*_ were the attributes that were most strongly correlated to the principal component analysis (Tables A–C in S1 File). Clusters in the final four-cluster solution were distinct (silhouette widths 0.24–0.60; mean width 0.41; Fig 2). The cluster group assigned to each CHaMP site is shown in Fig 3D and Fig G in S1 File.

### Comparison of framework agreement

**Fig 5:**
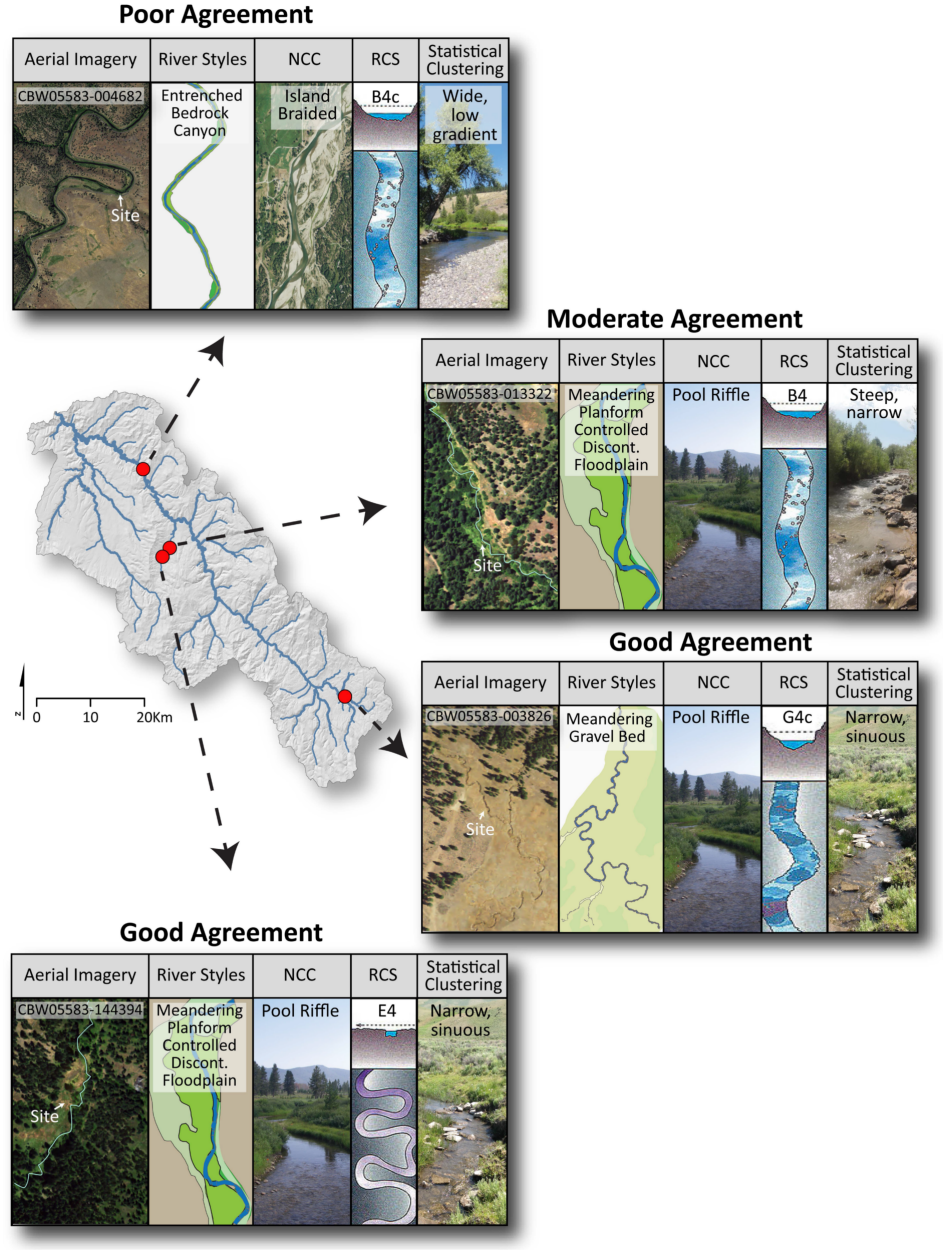
Illustrative example reaches describing agreement between classification outputs. Reaches at which the four classifications had poor agreement, moderate agreement, and good agreement in the observed channel planform.

**Fig 6:**
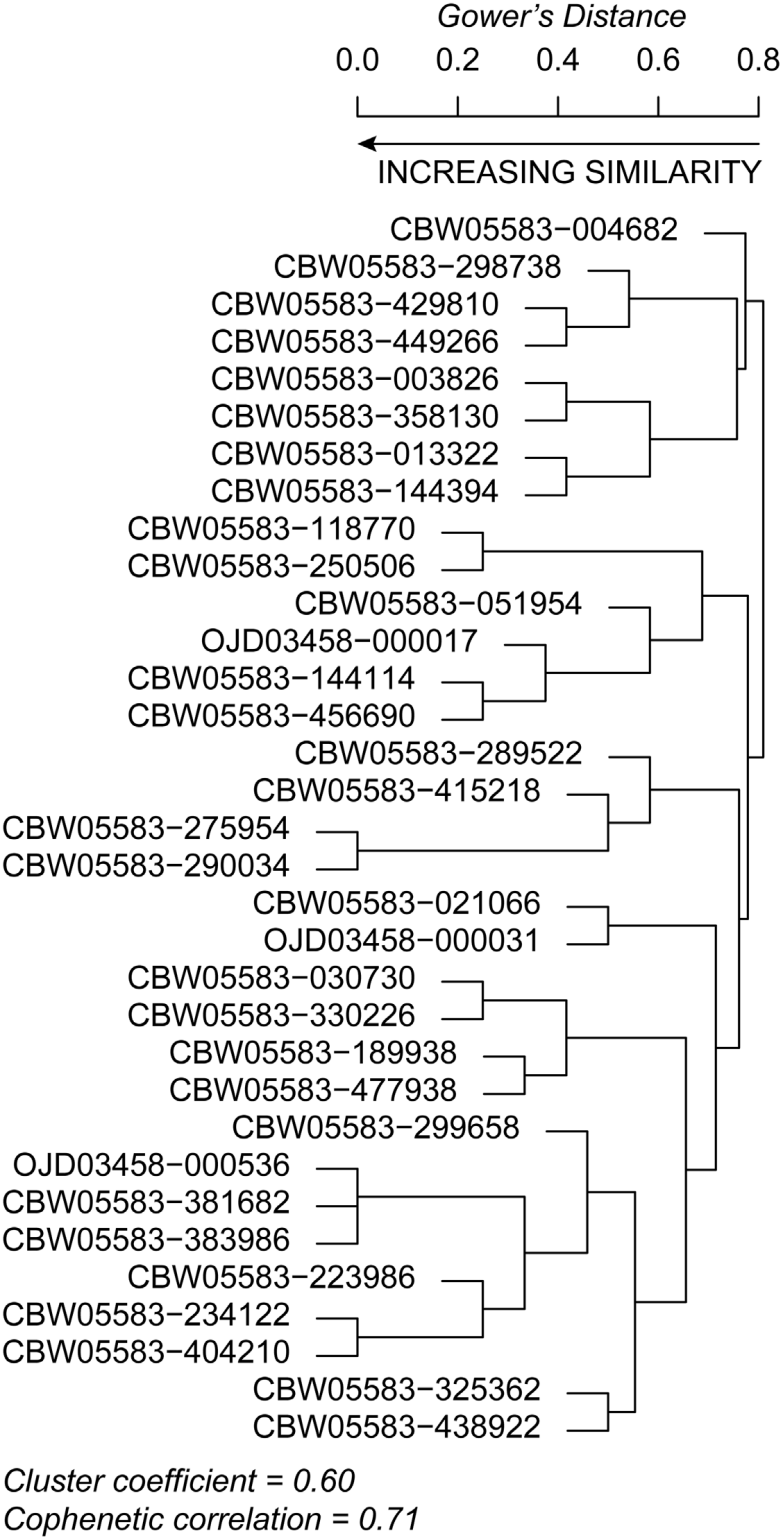
Dendrogram of clustered reaches based on their classification outputs from each of the four frameworks. Reaches with a distance of zero that occur on adjacent nodes of the same length are identical. For example, reaches CBW05583-381682 and CBW05583-383986 are identical in how they were classified by all four frameworks. Reaches were clustered using an average linkage clustering algorithm and Gower’s distance matrix.

**Fig 7:**
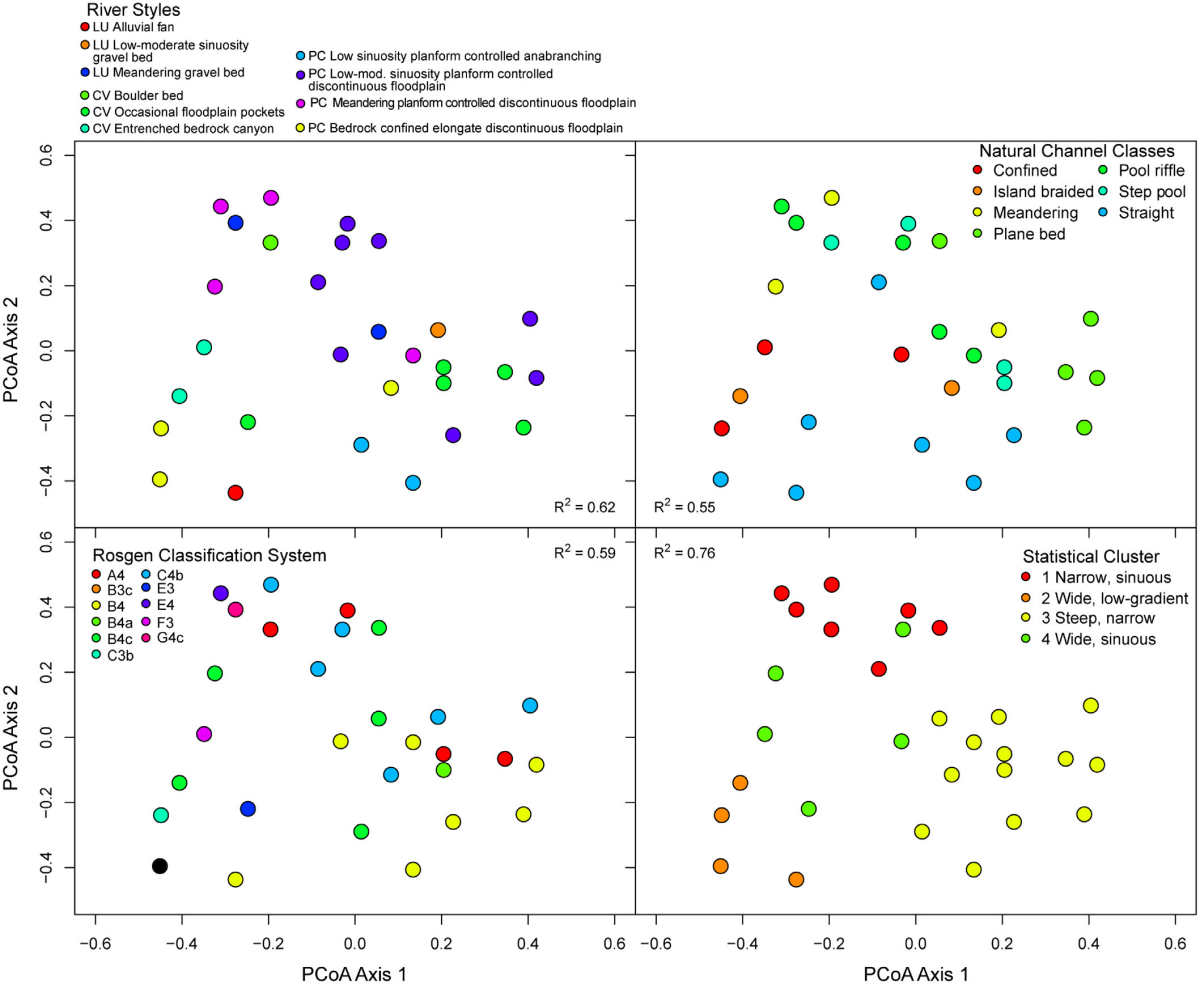
Principal coordinates analysis ordination showing reaches’ relative similarity based on the outputs of the four classification frameworks. Each reach is plotted within each classification output for ease of interpretation. Reaches were grouped within the ordination space using Gower’s distance. Reaches that are more similar to one another are closer together in the ordination space. R^2^ values correspond to the fit of a given classification framework’s outputs to the ordination of all classification outputs.

The analysis of agreement between reach types of each framework (Section 2.8; Table 5) generally indicates that far more often than not, frameworks produced reach type classifications that were congruent with one another. Clustering of the classification outputs at each reach showed that seven reaches had perfect agreement with at least one other reach; that is, each of the four classification outputs were identical across the four classifications (Table 5; Fig 5; distance of 0 in Figs 6 and 7). Seven reaches’ outputs agreed for three of the four outputs (distance of 0.2 in Fig 6). A majority of the reaches agreed on two of the four classification outputs (Table 5; Figs 6 and 7). Very few reaches classified as a combination of vastly different classification outputs than the other streams (Fig 6). These trends were apparent in the PCoA ordination of reaches based on their classification outputs (Fig 7). Similarly, when using expert judgment to compare the level of agreement between NCC and each of the other three frameworks at 33 CHaMP sites (for a total of 99 comparisons; Table 5), we found “good” agreement at 60 sites (61%), “moderate” agreement at 19 sites (19%), and “poor” agreement at 20 sites (Table 5). Thus, reasonable agreement (good or moderate) was found at 80% of sites.

**Table 5:** Classification results and agreement for each CHaMP site across the four classification frameworks.

CHaMP Site ID	Stream name	UTM Easting	UTM Northing	River Style Valley Confinement	River Styles	Natural Channel Classes	Rosgen Class. System	Statistical Clustering	Agreement
CBW05583-250506	Lunch Creek	377638	4930916	CV	Boulder Bed	Step Pool	A4	Narrow, sinuous	RS: Good; RCS: Mod; Cluster: Good
CBW05583-004682	Middle Fork John Day River	333505	4971313	CV	Entrenched Bedrock Canyon	Island Braided	B4c	Wide, low-gradient	RS: Poor; RCS: Poor; Cluster: Poor
CBW05583-021066	Middle Fork John Day River	337657	4968709	CV	Entrenched Bedrock Canyon	Confined	F3	Wide, sinuous	RS: Good; RCS: Good; Cluster: Mod.
CBW05583-144114	Vinegar Creek	380932	4942422	CV	Floodplain Pockets	Step Pool	A4	Steep, narrow	RS: Mod.; RCS: Mod.; Cluster: Good
CBW05583-223986	Bridge Creek	379613	4935524	CV	Floodplain Pockets	Plane Bed	B4	Steep, narrow	RS: Good; RCS: Good; Cluster: Good
CBW05583-456690	Butte Creek	369488	4942756	CV	Floodplain Pockets	Plane Bed	A4	Steep, narrow	RS: Good; RCS: Good; Cluster: Good
OJD03458-000017	West Fork Lick Creek	357991	4940711	CV	Floodplain Pockets	Step Pool	B4a	Steep, narrow	RS: Mod.; RCS: Good; Cluster: Good
CBW05583-051954	Dry Fork Clear Creek	383698	4934662	CV	Floodplain Pockets	Straight	E3	Wide, sinuous	RS: Good; RCS: Poor; Cluster: Poor
CBW05583-189938	Granite Boulder Creek	369068	4945617	LUV	Alluvial Fan	Straight	B4	Wide, low-gradient	RS: Mod.; RCS: Good; Cluster: Good
CBW05583-449266	Middle Fork John Day River	376782	4941104	LUV	Low-Moderate Sinuosity Gravel Bed	Meandering	C4b	Steep, narrow	RS: Mod.; RCS: Good; Cluster: Poor
CBW05583-003826	Summit Creek	386503	4937885	LUV	Meandering Gravel Bed	Pool Riffle	G4c	Narrow, sinuous	RS: Good; RCS: Good; Cluster: Good
CBW05583-358130	Squaw Creek	388721	4936107	LUV	Meandering Gravel Bed	Pool Riffle	B4c	Steep, narrow	RS: Good; RCS: Mod.; Cluster: Poor
CBW05583-289522	Middle Fork John Day River	378688	4939623	PC	Bedrock-controlled Elongate Discont. Floodplain	Island-Braided	C4b	Steep, narrow	RS: Poor; RCS: Poor; Cluster: Poor
CBW05583-275954	Middle Fork John Day River	364436	4947549	PC	Bedrock-controlled Elongate Discont. Floodplain	Straight	B3c	Wide, low-gradient	RS: Mod.; RCS: Good; Cluster: Good
CBW05583-290034	Middle Fork John Day River	370912	4944299	PC	Bedrock-controlled Elongate Discont. Floodplain	Straight	B3c	Wide, low-gradient	RS: Mod.; RCS: Good; Cluster: Good
CBW05583-415218	Middle Fork John Day River	361529	4948510	PC	Bedrock-controlled Elongate Discont. Floodplain	Confined	C3b	Wide, low-gradient	RS: Poor; RCS: Mod.; Cluster: Mod.
CBW05583-030730	Camp Creek	352247	4942752	PC	Low-Moderate Sinuosity Planform-Controlled Discontinuous Floodplain	Straight	B4	Steep, narrow	RS: Good; RCS: Good; Cluster: Good
CBW05583-330226	Camp Creek	357015	4947826	PC	Low-Moderate Sinuosity Planform-Controlled Discontinuous Floodplain	Straight	B4c	Steep, narrow	RS: Good; RCS: Good; Cluster: Good
CBW05583-118770	North Fork Bridge Creek	375925	4933066	PC	Low-Moderate Sinuosity Planform-Controlled Discontinuous Floodplain	Step Pool	A4	Narrow, sinuous	RS: Poor; RCS: Mod.; Cluster: Mod.
CBW05583-299658	Clear Creek	382042	4930368	PC	Low-Moderate Sinuosity Planform-Controlled Discontinuous Floodplain	Plane Bed	B4c	Narrow, sinuous	RS: Good; RCS: Good; Cluster: Mod.
CBW05583-438922	Dry Fork Clear Creek	384597	4933274	PC	Low-Moderate Sinuosity Planform-Controlled Discontinuous Floodplain	Straight	C4b	Narrow, sinuous	RS: Poor; RCS: Poor; Cluster: Poor
CBW05583-234122	Clear Creek	382238	4929332	PC	Low-Moderate Sinuosity Planform-Controlled Discontinuous Floodplain	Plane Bed	B4	Steep, narrow	RS: Good; RCS: Good; Cluster: Good
CBW05583-381682	Vinegar Creek	380718	4944390	PC	Low-Moderate Sinuosity Planform-Controlled Discontinuous Floodplain	Plane Bed	C4b	Steep, narrow	RS: Good; RCS: Good; Cluster: Good
CBW05583-383986	Camp Creek	353774	4936398	PC	Low-Moderate Sinuosity Planform-Controlled Discontinuous Floodplain	Plane Bed	C4b	Steep, narrow	RS: Good; RCS: Good; Cluster: Good
CBW05583-404210	Vinegar Creek	379442	4940614	PC	Low-Moderate Sinuosity Planform-Controlled Discontinuous Floodplain	Plane Bed	B4	Steep, narrow	RS: Good; RCS: Good; Cluster: Good
CBW05583-477938	Clear Creek	381713	4935379	PC	Low-Moderate Sinuosity Planform-Controlled Discontinuous Floodplain	Straight	B4	Steep, narrow	RS: Poor; RCS: Good; Cluster: Good
OJD03458-000536	Vinegar Creek	378654	4940187	PC	Low-Moderate Sinuosity Planform-Controlled Discontinuous Floodplain	Plane Bed	C4b	Steep, narrow	RS: Good; RCS: Good; Cluster: Good
CBW05583-325362	Summit Creek	390544	4937077	PC	Low-Moderate Sinuosity Planform-Controlled Discontinuous Floodplain	Pool Riffle	C4b	Wide, sinuous	RS: Mod.; RCS: Good; Cluster: Good
OJD03458-000031	Camp Creek	351579	4940332	PC	Low-Moderate Sinuosity Planform-Controlled Discontinuous Floodplain	Confined	B4	Wide, sinuous	RS: Poor; RCS: Mod.; Cluster: Poor
CBW05583-144394	Slide Creek	344959	4955342	PC	Meandering Planform-Controlled Discontinuous Floodplain	Pool Riffle	E4	Narrow, sinuous	RS: Good; RCS: Good; Cluster: Good
CBW05583-429810	Summit Creek	387760	4937802	PC	Meandering Planform-Controlled Discontinuous Floodplain	Meandering	C4b	Narrow, sinuous	RS: Good; RCS: Good; Cluster: Mod.
CBW05583-013322	Slide Creek	345607	4957140	PC	Meandering Planform-Controlled Discontinuous Floodplain	Pool Riffle	B4	Steep, narrow	RS: Good; RCS: Mod.; Cluster: Poor
CBW05583-298738	Middle Fork John Day River	385006	4938373	PC	Meandering Planform-Controlled Discontinuous Floodplain	Meandering	B4c	Wide, sinuous	RS: Good; RCS: Poor; Cluster: Good

There were consistent relationships between site morphology and the level of classification agreement (good, moderate, or poor). In the qualitative analysis of classification agreement (Table 5), individual reaches classified into groups of similar morphologies within one framework sometimes failed to align with a comparable group under another classification framework. This pattern was most apparent in confined reach types that did not aggregate into consistent groups across statistical clusters, Rosgen Classification System types, and natural channel classes. For example, River Styles’ confined valley with occasional floodplain pockets were classified as all four statistical clusters, five different RCS reaches, and three NCC classes (Table 5; Fig 7). In contrast, partly confined channel types were more likely to be grouped into only one or two channel types from other classifications. For example, River Styles’ partly confined low-moderate sinuosity, planform-controlled discontinuous floodplain grouped into RCS types of C4b and B4, and NCC classes of plane bed or straight planform, and steep/narrow and narrow/sinuous statistical clustering classes.

Additionally, the partly confined low-sinuosity planform-controlled anabranching river style occurred exclusively as B4 RCS classes, straight, narrow statistical cluster, and straight NCC. The partly confined bedrock-controlled elongate discontinuous floodplain river style classified as slightly to moderately entrenched, moderate sinuosity RCS types (C, B channels), and wide, low-gradient clusters, but was less consistently grouped by NCC (straight, confined, and island braided). While strict fidelity between groups within each classification did not occur, partly confined River Styles grouped well with the other classifications based on their component inputs.

## Discussion

### Comparison of classification outputs: example sites

Here we highlight four example sites to illustrate the varying degrees of framework agreement found during our classification output comparison. An example of poor agreement between the four frameworks was found at a confined valley reach on the Middle Fork John Day River (CHaMP site: CBW05583-004682), we found a B4c RCS type, wide, low-gradient statistical cluster, island-braided NCC, and entrenched bedrock canyon river style (Figs 5 and 6). Readers are referred to the supporting information (Fig F in S1 File) for the characteristics of each RCS channel type. The statistical classification matched the definition of a wide, low-gradient, B4c RCS channel type. While it is plausible that a B4c RCS channel type and an entrenched bedrock canyon river style could be applied to the same reach, the island-braided NCC classification is deserving of further exploration as it may hint at a departure from historic channel condition, which NCC attempts to predict. Subsequent field visits to this site by *O’Brien* [Personal Communication] note the presence of numerous legacy sediment deposits (e.g. [87]) above the active channel, within a wide valley bottom that allows for channel adjustment. These observations may imply that the system was overwhelmed by sediment during the early Holocene. Accordingly, the pre-disturbance classification of an island-braided channel using NCC may be appropriate in this case, and could hint at the background morphology of the channel.

In contrast, we found good agreement between all classification frameworks at two of the four example reaches (Fig 5) and seven total reaches (Figs 6 and 7). The first is a laterally unconfined reach on the Middle Fork John Day River (Fig 5; CHaMP site: CBW05583-003826) classified as a G4c RCS type, narrow sinuous statistical cluster, pool-riffle NCC, and meandering gravel bed river style. The second site is a partly confined reach on Slide Creek (Fig 5; CHaMP site: CWB05583-144394), classified as a meandering planform-controlled discontinuous floodplain river style. This site was further classified as an E4 RCS reach, pool riffle RCC type, and narrow, sinuous statistical cluster. At these locations, the combination of geomorphic characteristics produced a reach classification that was highly similar in terms of valley setting, planform, and assemblage of geomorphic units between all four frameworks. In the case of the former site, the reach occurs within a broader ~10 km segment of the Middle Fork John Day that exhibits a sinuous planform in an unconfined valley. The latter site also occurs in a ~5 km segment of Slide Creek that exhibits a consistent meandering planform. These more longitudinally-continuous reaches are undoubtedly helpful for agreement in classification among continuous frameworks (e.g. RSF and NCC) that may use disparate spatial scales of data (e.g. NHD+ and field-based validation versus NHD and basin-scale 10 m DEMs, respectively) and derive classifications remotely prior to field-based verification.

An example of a site with moderate agreement was found in a partly-confined valley setting on Slide Creek (Figs 5 and 6; CHaMP Site CBW05583-013322). This reach showed different, but plausible combinations of channel types. The reach was classified as a partly-confined valley with meandering planform-controlled discontinuous floodplain river style—whose in-channel geomorphic unit assemblage is essentially repeating pool-riffle sequences—and pool-riffle in NCC, but was classified as a B4 RCS and steep, narrow statistical cluster. Reaches like this that exhibit mixed agreement between classification frameworks highlight that subtle differences in channel form, such as channel gradient and sinuosity, can lead to significant differences in the classification of an individual reach. These differences arise as a result of the hierarchical and statistical clustering classifications used here, as the order of appearance of geomorphic metrics in a decision tree can vary between frameworks and subsequently affect classification output.

### Why do classification frameworks differ?

Differences in classification frameworks’ outputs ultimately arise because each framework emphasizes physical variables differently throughout the classification process. Although the data requirements between classification frameworks are similar, including channel planform metrics, substrate, and the ability of a channel to migrate and access sediment sources (Table 2), the order in which these attributes appear within a particular framework’s decision tree may vary markedly (see Figs B–F in S1 File). For example, at the broad planform scale, the first step in the differentiation of reach types within the RCS is to distinguish between single- and multi-thread channels. In contrast, this characterization of channel planform is completed several steps later in the River Styles framework, which instead places the greatest importance on the degree of valley confinement. Both RCS and River Styles, however, make their final differentiation between stream types based on the bed material texture within a reach.

When considering statistical approaches such as NCC and statistical clustering, all physical attributes are used in the grouping algorithm, and hierarchical decision trees are foregone. Because most statistical classification techniques computationally determine which of the input variables are most important in differentiating stream types, ranking them accordingly, *a priori* importance is not placed upon a given variable. While variables can be weighted in clustering and machine-learning algorithms to emphasize the importance of specific processes, many classifications, like NCC’s support vector machine, instead use training data to fit algorithms before computing classes for a data set. This approach is limited not by what variable is perceived to be most important, but rather, by what training data are available from which to build a model.

Similar constraints exist on the clustering method used here, which can only group reaches for which data are available. In building representative statistical classifications, having spatially-balanced, randomized sampling is ideal [88]. Another key methodological consideration in using statistical classification approaches is that the number of classes is often determined by the strength of the fit between data and algorithm, and must be validated by expert judgment of the classified statistical groups and their geomorphic likelihood. Robust clustering was observed here with a relatively small number of classes (four), whereas the other three classification schemes had between eight and fourteen classes. Accordingly, parameter and cluster algorithm selection, data transformation or standardization can all influence how well data fits a given clustering algorithm, with consequences for whether geomorphically meaningful groups correspond to statistically grouped data.

More generally, the difference in the relative importance of each physical variable within a particular classification framework points to the form-process interactions that each classification method attempts to document or explain. Particularly in the hierarchical approaches (e.g. RSF, RCS), the order of appearance of variables in the classification has a large impact on the classification of an individual channel reach. Distinct differences are also evident when the original intent of the classification framework is considered. Some frameworks produce analyses of current reach type (e.g. RSF, RCS, statistical clustering), while others predict pre-disturbance or natural channel morphology (e.g. NCC). Differences in the temporal output of each framework may not be intuitive, but provide a critical context for interpreting and using the outputs derived [88].

Classification frameworks appeared to disagree most frequently in settings where channel form was incongruous relative to valley width. That is, narrow valleys typically contain narrow, straight streams. Conversely, in wide valleys, we expect to find relatively wide, freely meandering streams that occupy large portions of the valley bottom [6]. In locations where this is not the case, we note that channel classification frameworks exhibited strong disagreement in their output (Table 5; Fig 7). For example, the frameworks disagreed in relatively wide valleys where channels occupied a small area of the valley floor (sites CBW05583-004682, CBW05583-289522, and CBW05583-415218) or exhibited unusually low sinuosity despite flowing through a wide valley. In contrast, some channels exhibited moderate sinuosity despite flowing in very narrow valleys (e.g. site CBW05583-051954; Table 5). A number of factors can lead to these scenarios, including anthropogenic straightening of channels to reduce bank erosion or lateral migration [89, 90], livestock grazing [91], or in the case of sinuous channels in narrow valleys, the presence of jams or dams associated with large woody debris accumulation and beaver activity, respectively [65,92]. The frameworks used here incorporate valley setting to draw inference on channel sinuosity, slope, and ultimately, the form of the channel. Particularly in NCC and RSF, valley setting is a top-level discriminator of channel classification (Figs B–E in S1 File). Components of RCS similarly leverage valley setting to infer the stream types found there [26,27]. Thus, we caution that despite the overall agreement between frameworks that we observed, classification outputs may differ markedly in locations where geomorphic, biotic, or anthropogenic factors cause channels to diverge from expected forms given a particular valley setting. We note that further research is needed to assess classification agreement in areas where valley setting may not be a reliable predictor of channel form (e.g., ephemeral channels, channels at the meandering/braided transition, and in heavily disturbed watersheds).

### Form and process in channel classification

Our comparison of four distinct classification frameworks demonstrates that there is significant overlap and agreement between outputs of the classifications used here. The most common result in all four frameworks was some variant of moderately-high gradient channel, in a partly-confined valley setting, with coarse gravel substrate, reflecting the high relief nature of the Middle Fork John Day Basin resulting from resistant igneous and metamorphic lithologies (Fig 3). Similarly, the least common channel types in all four frameworks were those variants corresponding to wide, freely meandering, low-gradient streams. These laterally unconfined streams are the ones most emphasized in classic channel planform classification and the fluvial geomorphology literature [93], although they are rare in many montane regions [34].

The four classification frameworks showed widespread agreement between their outputs despite being variably based in either form or process (Table 2). While all four frameworks contained metrics that either directly described the processes at work in channel reaches or employed measurements of channel form as surrogates for geomorphic processes, the relative role of form- and process-based components varied between frameworks. For example, while the RSF depends on observation of processes (e.g. channel behavior at overbank flow, interaction with vegetation), NCC and RCS rely on measurements of channel form that are directly related to sediment supply and transport competence at individual channel reaches. Taken to the extreme, the statistical clustering approach used here exclusively relies on field-based measures of channel form in an attempt to differentiate individual reaches. Despite the range of form- and process-based metrics in each framework, the four approaches exhibited overall agreement, suggesting that a simple differentiation in terms of form or process does not characterize the utility of a particular approach.

When considering how the geomorphic community groups classification frameworks (see [3]), the line between those based in form and those based in process is not necessarily clear. Many common stream classification frameworks defy such simple binning, instead combining aspects of form and process to group river reaches. In general, the use of channel form metrics as surrogates for stream or valley-scale processes is widespread [3]. This is perhaps a reflection of the complexity involved in a purely process-based classification framework, which would require high-resolution measurement of rates of sediment transport, supply, and channel adjustment at many sites throughout a stream network [4]. Such approaches are only possible under exceptional mandates that require a great deal of human and financial capital (e.g. [94]). In most basins, classification frameworks based on channel form metrics are practical surrogates for inferring process.

Similarly, rapid geomorphic assessment (RGA) methods that nominally characterize process domains along streams often rely on form-based measurements or observations (e.g. degree of bank erosion or channel incision) to infer processes related to channel stability [10]. We acknowledge that the degree to which cutoffs and thresholds between channel types, particularly in hierarchical classification schemes (e.g. RSF, NCC, RCS), reflect true transitions in process domains likely requires further investigation. At the same time, form-based assessments have been borne of a necessity to characterize river reaches over large spatial scales within a reasonable timeframe and at moderate costs, leading to their widespread application in the geomorphic, land management, and hydrologic communities.

While classifications represent “snapshots” of reaches, rivers are dynamic and adjust in response to water and sediment supply [95,96]. If these boundary conditions are not considered, assumptions of stability may be made when channel form may actually indicate a transient, or responding state, given altered sediment or water availability. For this reason, some classification frameworks separate current character and behavior from past evolution, condition and trajectory (e.g. the RSF), and others separate condition (e.g. RCS). In other systems, the degree of channel departure from background conditions is considered and may completely invalidate certain frameworks. For example, in watersheds heavily influenced by mill dams or beaver ponds and their associated legacy sediment deposits [87,97], the NCC classification approach may not provide an informative river classification as this method predicts pre-disturbance channel planform.

There are instances where applying multiple classification frameworks may yield insight into channel processes or watershed disturbance history that individual frameworks may not reveal. For example, divergence between frameworks that classify current versus historic channel form (e.g., RSF and NCC, respectively) may point to reaches that have undergone significant disturbance and planform alteration, and thus differ from expected background characteristics. Alternatively, the comparison of watershed-scale frameworks with reach-based classifications (e.g., RSF versus RCS) may aid in pinpointing reaches that differ from the characteristic downstream progression of channel patterns within a basin, thus providing insight into local geologic/geomorphic controls or areas of intense disturbance that drive channel form.

Finally, it is worth exploring whether our use of a single watershed may have influenced the results of this study, particularly with regard to the performance of, and subsequent agreement between, the classification frameworks employed here. The MFJD basin was specifically chosen for this research for its geomorphic diversity: a wide range of bedrock and surficial geologies drive large gradients in elevation that correspond to variations in precipitation and climate, which in turn lead to a diversity of vegetation assemblages across the watershed [52]. Together, these biophysical gradients create a range of channel types that are reflected in the classification frameworks’ outputs used in our comparison: ten of fourteen possible river styles, seven of nine possible NCC classes, and six of seven possible RCS channel types were found at the 33 CHaMP sites used for direct comparison in this study. Overall, the agreement between classification outputs across a wide range of channel types gives us confidence that these frameworks will likely demonstrate similarly robust agreement when applied to basins other than the MFJD. At the same time, we observed that disagreement in the frameworks’ output was common when valley confinement was not a reliable predictor of the resulting channel form (e.g. narrow, straight channels in wide valleys; Table 5). As a result, basins that have undergone pervasive and widespread disturbance, leading to divergence between channel form and valley confinement (whether natural or anthropogenic; [97, 98]) may be particularly susceptible to classification disagreement, and we encourage similar classification comparisons in these settings.

## Conclusions

Stream classification schemes are widely used to make inference about how channel and floodplain landforms respond to hydrologic and geomorphic processes. In the absence of exhaustive sediment transport, hydrologic, and/or hydraulic data, and the budget and personnel to collect and analyze them, stream classification provides a critical tool for watershed managers to understand the range of potential reach types in a watershed and their likely driving processes. Here we have applied four distinct classifications, finding that each methodology is informative of the range of potential channel types within the Middle Fork John Day River Watershed. We assessed overall agreement between frameworks using both a qualitative, expert judgment-based approach, as well as a quantitative statistical intercomparison of outputs; both methods pointed to widespread agreement between framework output. Classification outputs were often highly comparable based on their component data inputs, and the four frameworks used here frequently output groups of reaches with similar hydrogeomorphic settings, distributions of in-channel geomorphic units, and bed sediment characteristics. Classification frameworks’ outputs diverged in locations where valley setting was not a reliable predictor of channel form.

The classifications used here range from thorough network based approaches (RSF, NCC) to more rapid reach-based approaches (statistical clustering, RCS) that categorize current (RCS, statistical clustering, RSF), historic (NCC; later stages of RSF not discussed here), and potential future trajectories of stream channels (later stages of RSF not discussed here). To varying degrees, they classify stream reaches based on aspects of process and form, yet the overall agreement between the frameworks indicates that, because process and form are intrinsically linked in rivers, form- and process-based approaches to assessing stream channel diversity are not mutually exclusive. In many cases, inference about fluvial dynamics can be readily drawn from measurements of physical channel characteristics.

## Supporting Information

S1 File Supporting text, figures, and tables for manuscript. (DOCX)
