# Supplementary material for: The Blurred Line between Form and Process: A Comparison of Stream Channel Classification Frameworks
Source: PLoS One. 2016 Mar 16;11(3):e0150293. doi: 10.1371/journal.pone.0150293 (PMC4794181; doi:10.1371/journal.pone.0150293)
Supplement: S1 File — (DOCX) [file pone.0150293.s001.docx]

Supporting Information for

# The Blurred Line between Form and Process: a Comparison of Stream Channel Classification Frameworks

Alan Kasprak*^,1,9^, Nate Hough-Snee*^,1,2^, Tim Beechie^3^, Nicolaas Bouwes^4^, Gary Brierley^5^, Reid Camp^1,4^, Kirstie Fryirs^6^, Hiroo Imaki^7^, Martha L. Jensen^1^, Gary O’Brien^1^, David L. Rosgen^8^, and Joseph M. Wheaton^1,2^

^1^Department of Watershed Sciences, Utah State University, Logan, UT 84322-5210, USA

^2^Ecology Center, Utah State University, Logan, UT, 84322-5210, USA

^3^Watershed Program, Fish Ecology Division, Northwest Fisheries Science Center, NOAA Fisheries, Seattle, WA 98112, USA

^4^Eco Logical Research, Providence, UT, 84332, USA

^5^School of Environment, University of Auckland, New Zealand

^6^Department of Environmental Sciences, Macquarie University, Sydney, Australia

^7^Pacific Spatial Solutions, Reston, VA, 20190, USA

^8^Wildland Hydrology, Fort Collins, CO, 80524, USA

^9^*Now at* U.S. Geological Survey, Grand Canyon Monitoring and Research Center, Flagstaff, AZ, 86001, USA

*These authors made equal contributions to the manuscript.

akasprak@usgs.gov; nate@natehough-snee

**Contents of this file**

Text A

Figures A to K

Tables A to C

**Introduction**

This supporting information contains descriptions of the methods and requisite datasets used to complete the River Styles Framework, Natural Channel Classification, the Rosgen Classification Framework, and statistical clustering used in the manuscript. It also contains graphical comparisons between the classification frameworks. Data used in the manuscript can be accessed at http://figshare.com/s/9e02827a92fc11e5adb406ec4b8d1f61. Note that any discrepancies between Natural Channel Classification in line and point data are the result of merging disparate linework datasets (NHD and NHD+) and are display artifacts only. Individual points have been checked for agreement with original NCC classification.

**Text A.** **Statistical Classification Methods and Results Supplement**

To classify streams of the John Day Basin, we used divisive clustering by partitioning around medoids to classify CHaMP reaches by their physical metrics. We opted to use divisive hierarchical clustering over hierarchical agglomerative clustering, because this approach initially takes into account the global distribution of the sample data. We grouped 33 unique stream reaches based on reach-level habitat attributes. A Euclidean distance matrix was calculated from the standardized data. This distance matrix was clustered into cluster configurations with 3-11 groups of reaches. These cluster solutions were assessed for their mean silhouette width and cluster uniqueness was verified using PERMANOVA models (Anderson, 2001) at an alpha of *P < 0.05*. The final cluster solution that we selected based on silhouette width and PERMANOVA models had four unique stream clusters. Clusters are summarized by channel attributes below in Table S.2. We validated channel attribute associations using principal components analysis (PCA) of the reach-level habitat attributes and fitting vectors of environmental variables over the PCA solution (Figure 3; Figure S5). We present the correlations between each channel form attribute and the principal components in Tables S.2 and S.3.


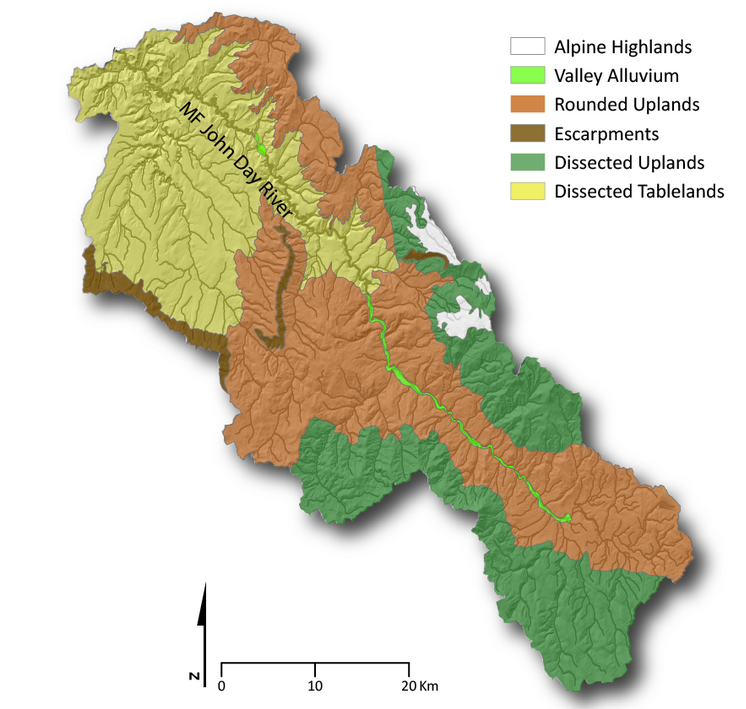


**Figure A. Landscape units delineated as an early step in the River Styles Framework of Brierley and Fryirs (2005) as employed by O’Brien and Wheaton (2015).**


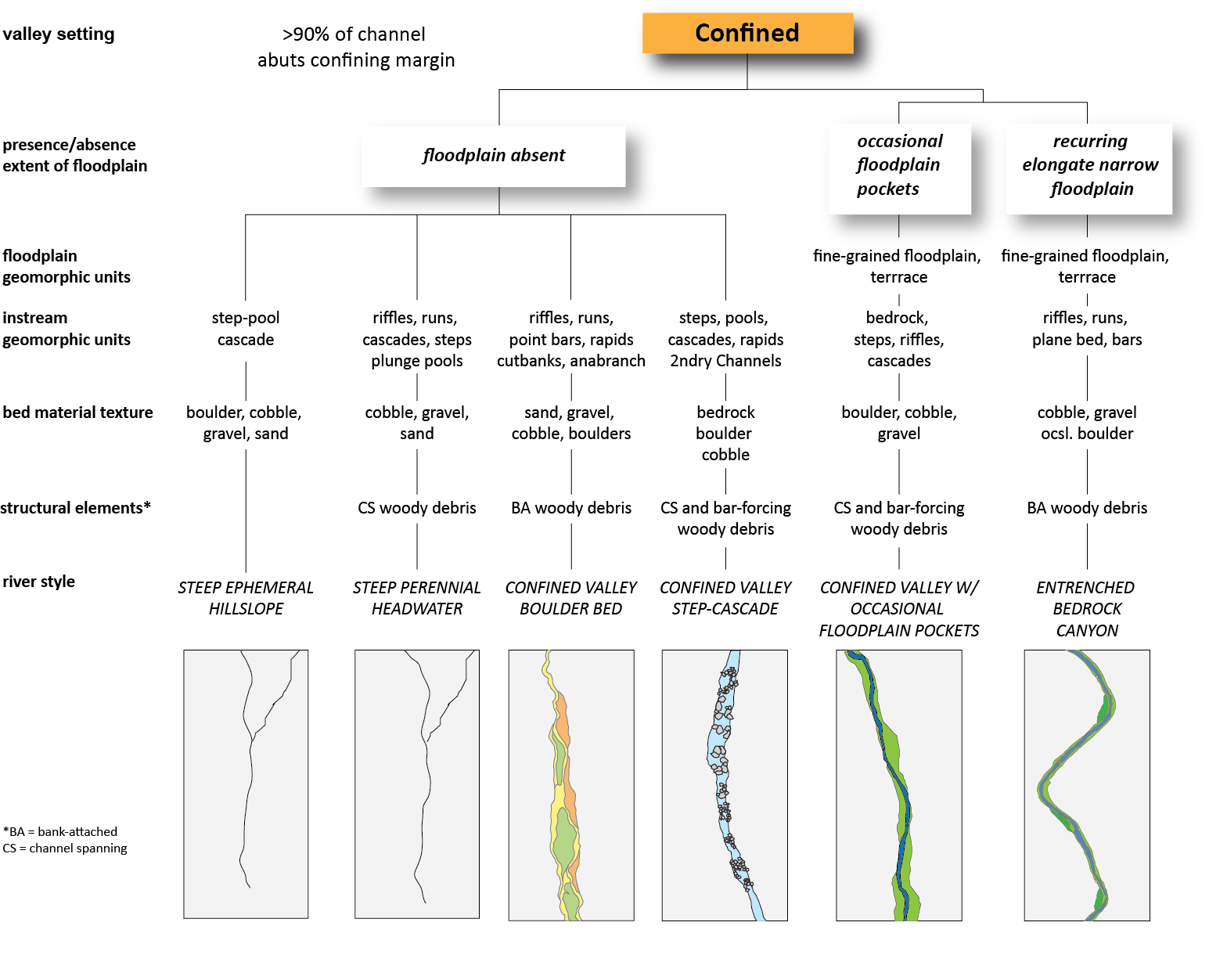


**Figure B. River styles tree used to determine reach type for confined channels. Figure from O’Brien and Wheaton (2015).**

**
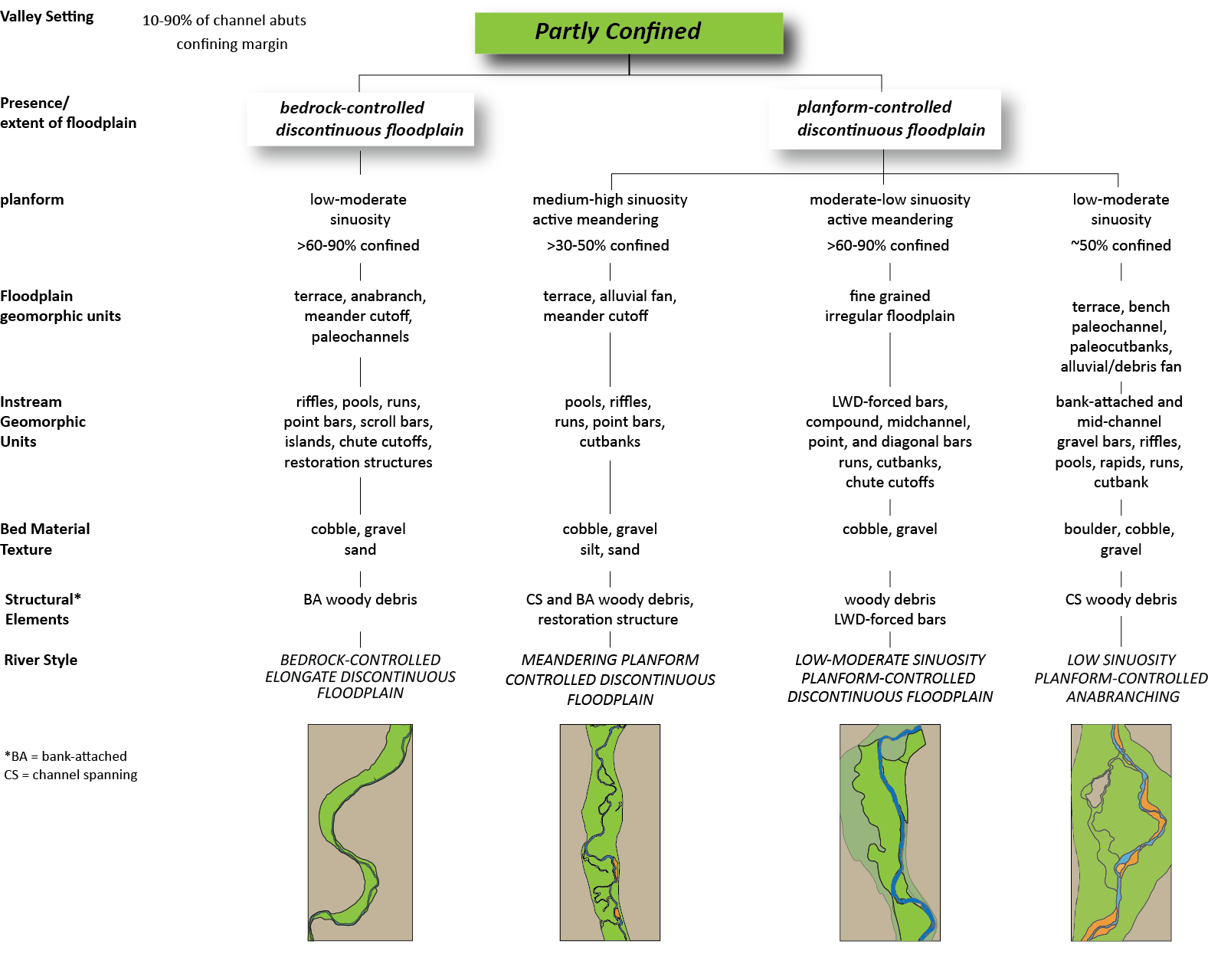
**

**Figure C. River styles tree used to determine reach types for partly confined channels. Figure from O’Brien and Wheaton (2015).**

**
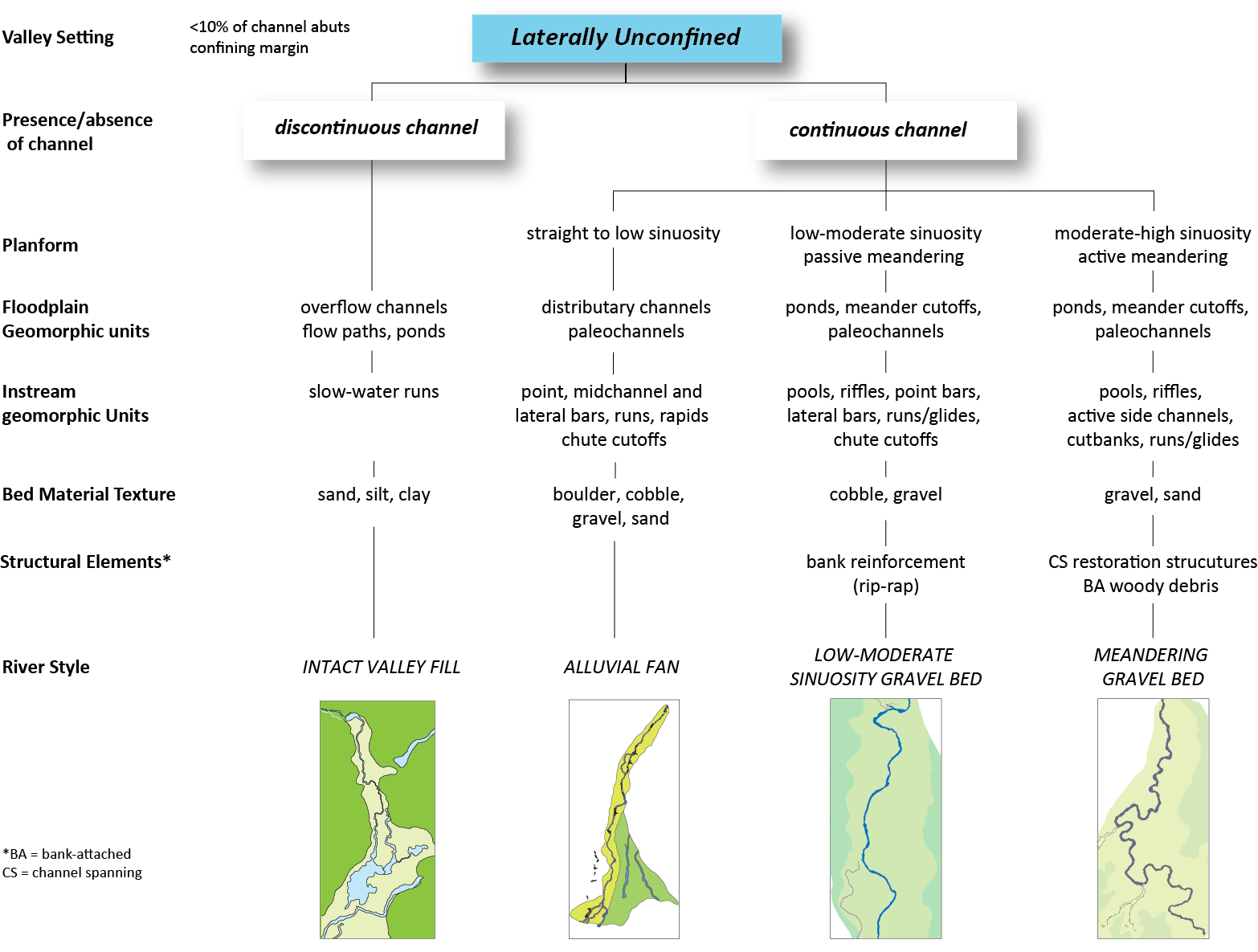
**

**Figure D. River styles tree used to determine reach types for laterally unconfined channels. Figure from O’Brien and Wheaton (2015).**


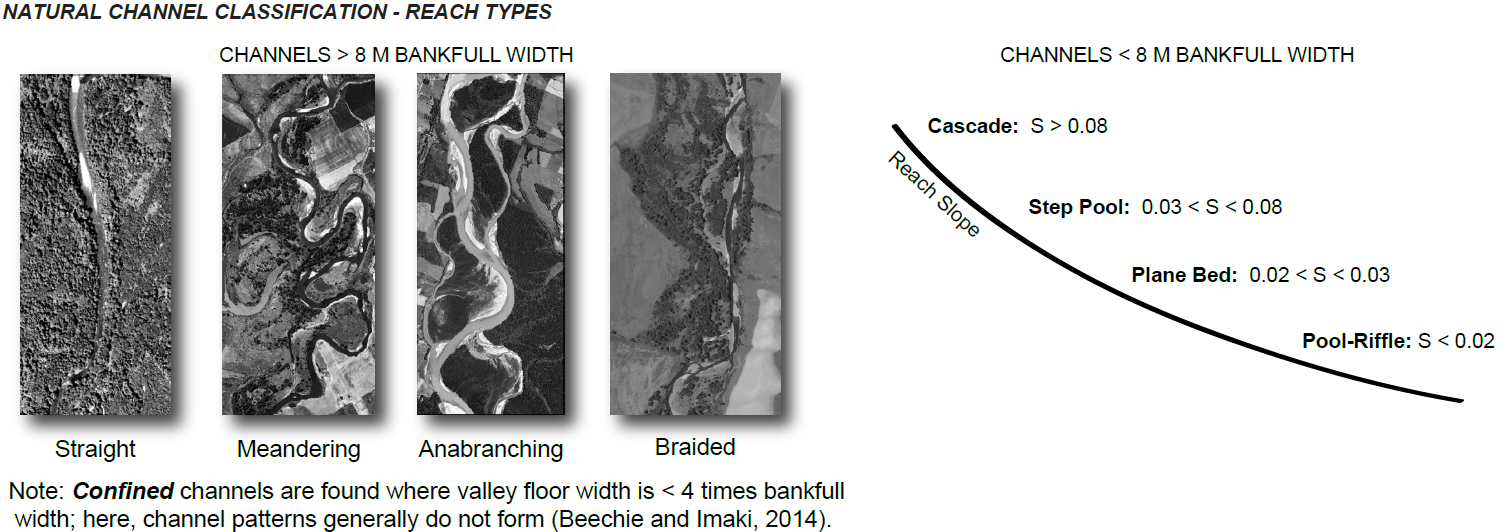


**Figure E. The Natural Channel Classification framework used in identifying historic planforms of the Middle Fork John Day Watershed for the entire watershed stream network and CHaMP reaches. Modified from Beechie and Imaki (2014).**


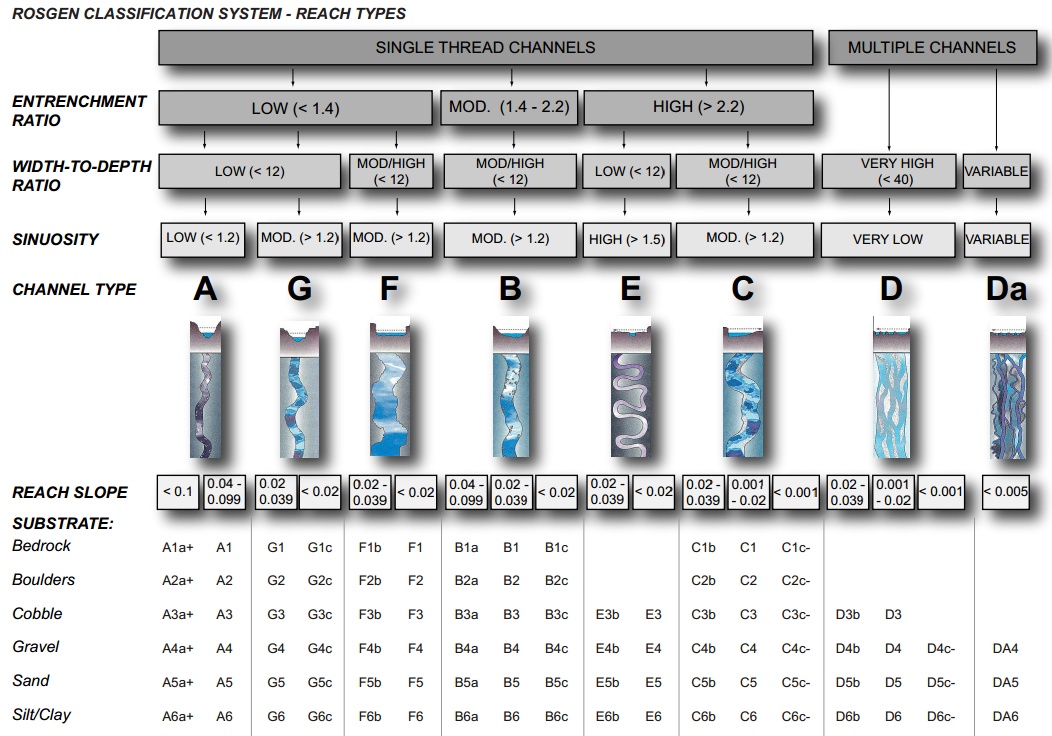


**Figure F. Hierarchical tree used in the Rosgen Classification System (Rosgen, 1994; Rosgen and Silvey, 1996) to determine reach types at CHaMP reaches of the Middle Fork John Day River watershed.**


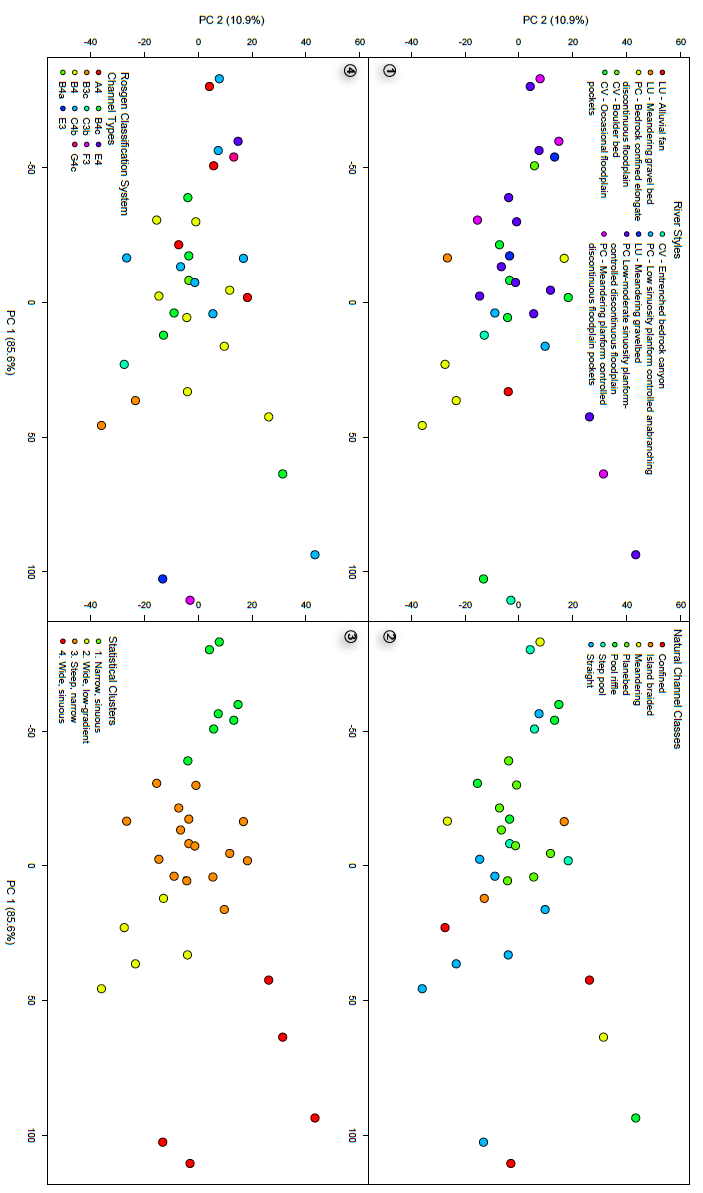


**Figure G. PCA Ordination of the 33 CHaMP reaches, plotted by classification results from each framework. Clockwise from top left: River Styles, Natural Channel Classification, Rosgen Classification System, and Statistical Classification.**

**
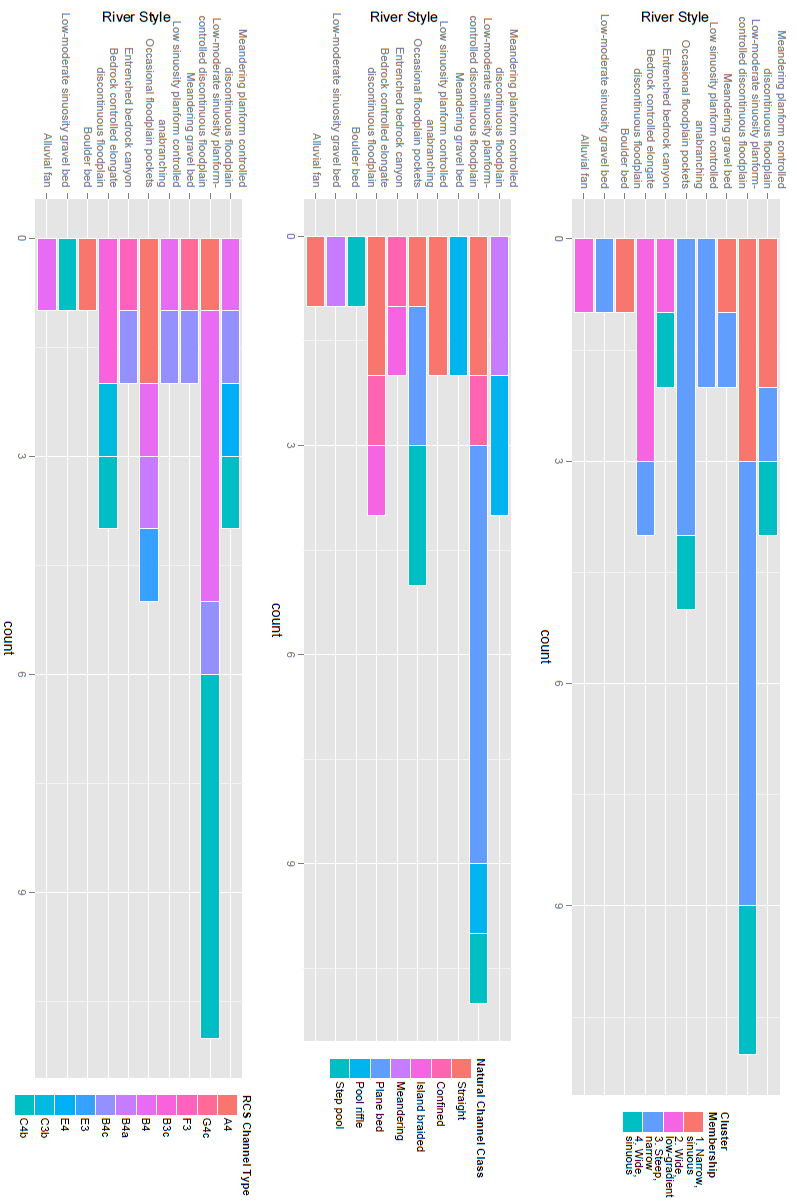
**

**Figure H. Histograms of the number of CHaMP reaches classified into each level of each classification framework, grouped by River Styles.** All classification level counts are presented from most confined (warm colors) to least confined (cool colors).

**
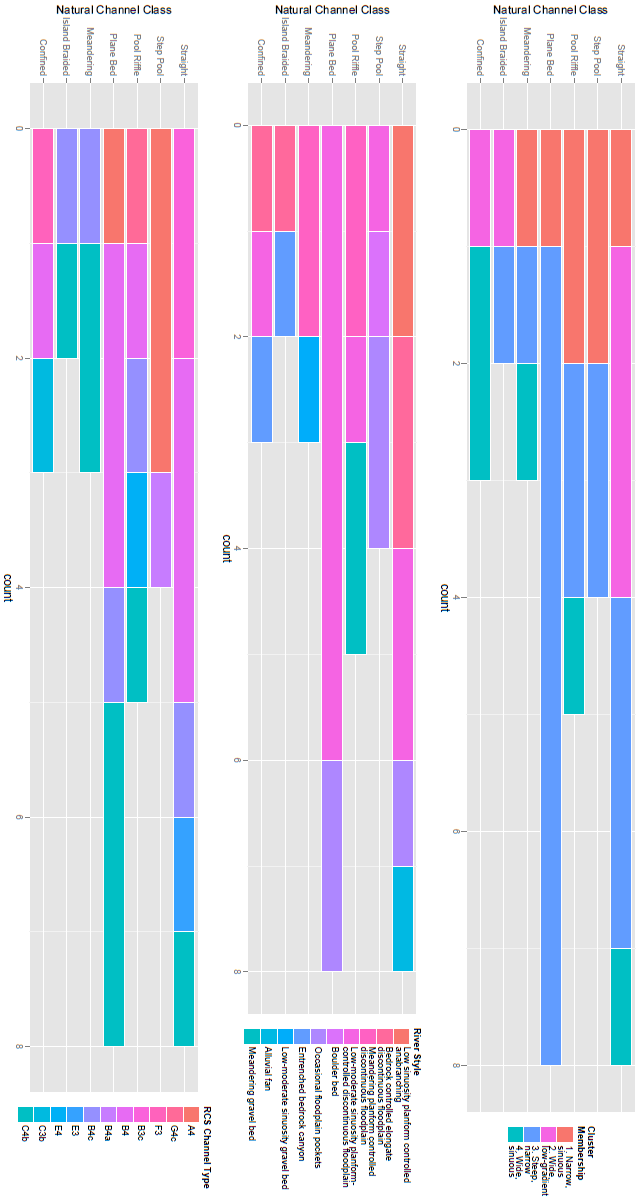
**

**Figure I. Histograms of the number of CHaMP reaches classified into each level of each classification framework, grouped by Natural Channel Classification.** All classification level counts are presented from most confined (warm colors) to least confined (cool colors)

.**
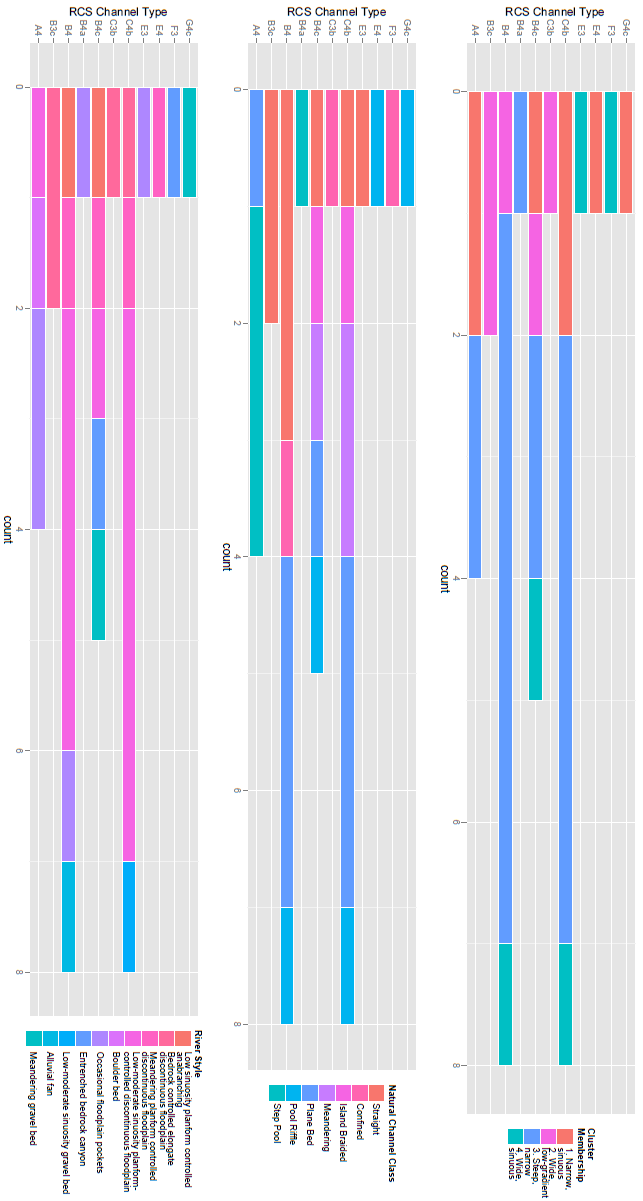
**

**Figure J. Histograms of the number of CHaMP reaches classified into each level of each classification framework, grouped by Rosgen Classification System.** All counts are presented from most confined (warm colors) to least confined (cool colors).

**
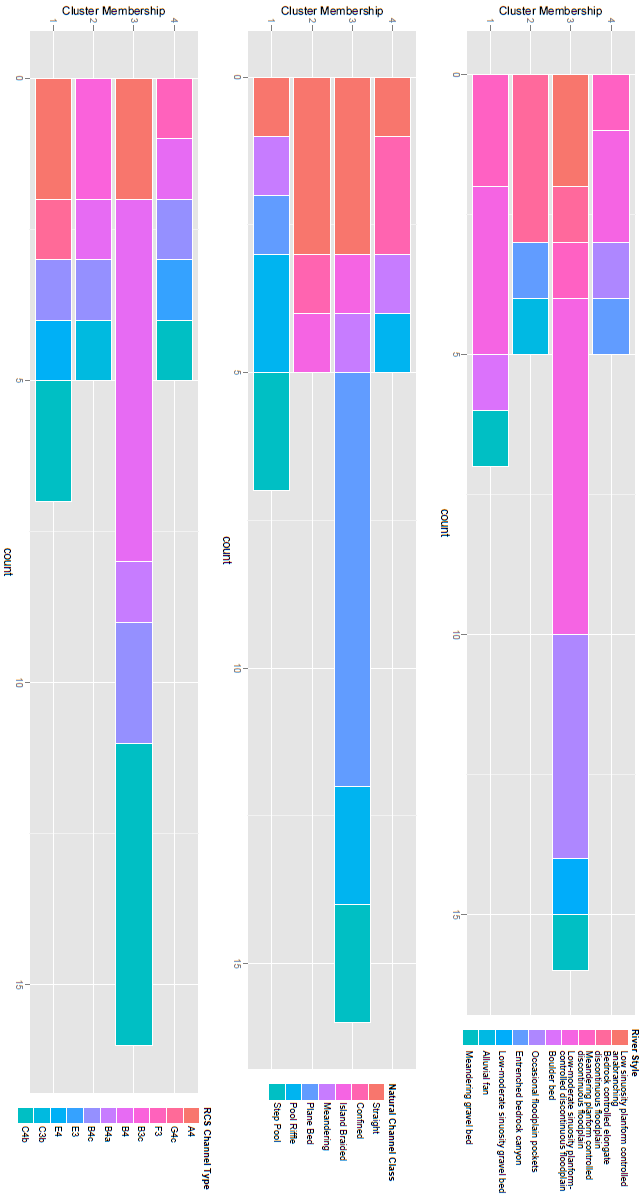
**

**Figure K. Histograms of the number of CHaMP reaches classified into each level of each classification framework, grouped by statistical clustering.** All counts are presented from most confined (warm colors) to least confined (cool colors).

**Table A. Summarized channel metrics for each cluster derived from partitioning around medoids.**

| Cluster | Bankfull width (m) | Sinuosity (%) | Gradient (%) | *D_16_* (mm) | *D_50_* (mm) | *D_84_* (mm) | Wetted  Width (m) | Bankfull width to depth ratio |
| --- | --- | --- | --- | --- | --- | --- | --- | --- |
| 1 | 2.82 | 1.13 | 1.50 | 5 | 26 | 61 | 2.52 | 14.75 |
| 2 | 18.1 | 1.15 | 0.54 | 41 | 67 | 125 | 10.17 | 32.35 |
| 3 | 6.40 | 1.18 | 1.79 | 18 | 49 | 97 | 3.78 | 23.20 |
| 4 | 8.62 | 1.07 | 1.28 | 9 | 40 | 182 | 5.35 | 26.89 |

Values are the mean value for each cluster.

**Table B. Principal component summary statistics include the PCA rotation for channel attributes (rows) by components (columns).**

| Metric | PC1 | PC2 | PC3 | PC4 | PC5 | PC6 | PC7 | PC8 |
| --- | --- | --- | --- | --- | --- | --- | --- | --- |
| Bankfull width | 0.063 | -0.133 | 0.599 | -0.451 | -0.476 | -0.207 | 0.382 | 0.027 |
| Sinuosity | -0.001 | 0.001 | -0.003 | -0.004 | -0.002 | 0.034 | 0.088 | -0.996 |
| Gradient | -0.005 | 0.016 | -0.060 | 0.025 | 0.057 | -0.946 | -0.307 | -0.059 |
| *D_16_* | 0.133 | -0.625 | -0.242 | 0.485 | -0.545 | -0.023 | 0.021 | 0.000 |
| *D_50_* | 0.286 | -0.690 | -0.052 | -0.397 | 0.530 | 0.013 | -0.006 | 0.000 |
| *D_84_* | 0.944 | 0.316 | -0.054 | 0.041 | -0.072 | 0.000 | -0.001 | -0.001 |
| Wetted width | 0.035 | -0.081 | 0.321 | -0.157 | -0.225 | 0.246 | -0.865 | -0.068 |
| Bankfull width to depth ratio | 0.072 | -0.093 | 0.686 | 0.614 | 0.369 | -0.024 | 0.052 | -0.001 |
| Standard deviation | 47.904 | 17.071 | 7.328 | 4.547 | 3.406 | 0.989 | 0.604 | 0.116 |
| Proportion  of variance explained | 0.858 | 0.109 | 0.020 | 0.008 | 0.004 | 0.000 | 0.000 | 0.000 |
| Cumulative proportion of variance explained | 0.858 | 0.967 | 0.987 | 0.995 | 0.999 | 1.000 | 1.000 | 1.000 |

The standard deviation, variance explained, and cumulative variance explained by each component are listed in bottom three rows.

**Table C. Structure correlations between principal components and channel attributes.**

| Metric | PC1 | PC2 | PC3 |
| --- | --- | --- | --- |
| Bankfull width | 0.471 | -0.358 | 0.691 |
| Sinuosity | -0.313 | 0.066 | -0.146 |
| Gradient | -0.23 | 0.246 | -0.388 |
| *D_16_* | 0.494 | -0.829 | -0.138 |
| *D_50_* | 0.75 | -0.646 | -0.021 |
| *D_84_* | 0.993 | 0.118 | -0.009 |
| Wetted width | 0.486 | -0.405 | 0.691 |
| Bankfull width to depth ratio | 0.494 | -0.225 | 0.717 |
